# A Chimeric Japanese Encephalitis Vaccine Protects against Lethal Yellow Fever Virus Infection without Inducing Neutralizing Antibodies

**DOI:** 10.1128/mBio.02494-19

**Published:** 2020-04-07

**Authors:** Niraj Mishra, Robbert Boudewijns, Michael Alexander Schmid, Rafael Elias Marques, Sapna Sharma, Johan Neyts, Kai Dallmeier

**Affiliations:** a KU Leuven Department of Microbiology, Immunology and Transplantation, Rega Institute, Laboratory of Virology and Chemotherapy, Molecular Vaccinology and Vaccine Discovery Group, Leuven, Belgium; University of Texas Medical Branch; University of Hong Kong

**Keywords:** flavivirus, chimeric YFV-17D vaccine, chimeric flavivirus vaccine, cross-protection, dual protection, antibody-dependent enhancement, nonneutralizing antibodies, antibody-dependent cellular cytotoxicity (ADCC), protective T cell responses, off-label use of vaccine

## Abstract

Recent outbreaks of yellow fever virus (YFV) in West Africa and Brazil resulted in rapid depletion of global vaccine emergency stockpiles and raised concerns about being unprepared against future YFV epidemics. Here we report that a live attenuated virus similar to the Japanese encephalitis virus (JEV) vaccine JE-CVax/Imojev that consists of YFV-17D vaccine from which the structural (prM/E) genes have been replaced with those of the JEV SA14-14-2 vaccine strain confers full protection in mice against lethal YFV challenge. In contrast to the YFV-17D-mediated protection against YFV, this protection is not mediated by neutralizing antibodies but correlates with YFV-specific nonneutralizing antibodies and T cell responses against cell-associated YFV NS1 and other YFV nonstructural (NS) proteins. Our findings reveal the potential of YFV NS proteins to mediate protection and demonstrate that chimeric flavivirus vaccines, such as Imojev, could confer protection against two flaviviruses. This dual protection may have implications for the possible off-label use of JE-CVax in case of emergency and vaccine shortage during YFV outbreaks. In addition, populations in Asia that have been vaccinated with Imojev may already be protected against YFV should outbreaks ever occur on that continent, as several countries/regions in the Asia-Pacific are vulnerable to international spread of the YFV.

## Abstract (precis)

Efficient and safe vaccines against yellow fever (e.g., YFV-17D) that provide long-lasting protection by rapidly inducing neutralizing antibody responses exist. However, the vaccine supply cannot cope with an increasing demand posed by urban outbreaks in recent years. Here we report that JE-CVax/Imojev, a YFV-17D-based chimeric Japanese encephalitis vaccine, also efficiently protects against YFV infection in mice. In case of shortage of the YFV vaccine during yellow fever outbreaks, (off-label) use of JE-CVax/Imojev may be considered. Moreover, wider use of JE-CVax/Imojev in Asia may lower the risk of the much-feared YFV spillover to the continent. More generally, chimeric vaccines that combine surface antigens and replication machineries of two distinct flaviviruses may be considered dual vaccines for the latter pathogen without induction of surface-specific antibodies. Following this rationale, novel flavivirus vaccines that do not hold a risk for antibody-dependent enhancement (ADE) of infection (inherent to current dengue vaccines and dengue vaccine candidates) could be designed.

## INTRODUCTION

Several flaviviruses, such as the yellow fever virus (YFV), Japanese encephalitis virus (JEV), dengue virus (DENV), Zika virus (ZIKV), West Nile virus (WNV), and tick-borne encephalitis virus, are important human pathogens. Flaviviruses are spread worldwide, though some species show a pronounced restriction to defined regions of endemicity, such as YFV to sub-Saharan Africa and tropical Latin America and JEV to Southeast Asia and the Asia-Pacific. Certain flaviviruses, such as DENV, WNV, and, most recently, ZIKV, are (re-)emerging in new areas (1–3). Some evidence suggests the first autochthonous transmission of JEV in Africa (4).

Yellow fever (YF) is an acute viral hemorrhagic disease which is currently endemic to ∼50 countries with ∼1 billion people living at risk of infection. Despite the availability of a highly efficient vaccine (YFV-17D; e.g., Stamaril), an estimated ∼0.2 million YFV infections with 29,000 to 60,000 deaths occur annually (5). Recent YFV outbreaks in Angola (2015-2016), the Democratic Republic of the Congo (2016), Brazil (2017), and Nigeria (2018) and a shortage of the YF vaccine supply raised serious concerns about the preparedness for future outbreaks (6, 7). Since the Aedes aegypti mosquito, the main YFV vector, is omnipresent in (sub)tropical Asia, YFV spillover to Asia and the establishment of epidemics involving urban transmission become increasingly realistic (8, 9).

For JEV, several licensed inactivated and live attenuated vaccines, including Ixiaro (inactivated vaccine) and JE-CVax (Imojev; YFV-17D-based chimeric live attenuated vaccine [c-LAV]), are available (10, 11). Another YFV-17D-based tetravalent c-LAV, namely, against dengue (CYD-TDV, Dengvaxia), has reached marketing licensure and is being introduced in some countries/regions. However, there are serious concerns related to the use of this vaccine in dengue virus-seronegative individuals, mainly because of aggravation of dengue disease by preexisting antibodies (antibody-dependent enhancement [ADE]) of DENV infection (12–14).

Vaccination against flaviviruses generally relies on the strategy to mount protective humoral immunity against structural proteins, in particular neutralizing antibodies (nAbs) elicited against the viral envelope (E) protein (5, 15), though also CD4^+^ T cells seem to contribute to the protective activity of current YFV vaccines (16). Nonetheless, experimental evidence obtained with mice and nonhuman primates for YFV (17–20) and, more recently, in mice also for WNV (21) and ZIKV (22) clearly shows that also nonstructural (NS) proteins, in particular NS1, can evoke protective humoral and cellular immune responses. Of note, NS1 is not part of the infectious flavivirus particle and thus not a target of nAbs. Likewise, immunization with an adenovirus vector encoding the NS3 protein of YFV-17D elicited strong CD8^+^ T cell responses, which resulted in some degree of protection in mice against subsequent challenge (23). However, full protection was observed only when the vaccine included the structural proteins of YFV-17D as the antigen as well (23, 24), obviously in line with the accepted role nAbs play in YFV infection. Thus, besides humoral immune responses against the E protein, cellular immune responses against the NS proteins may to some extent also contribute to immunity against flaviviruses. However, no flavivirus vaccines that are based on any of these NS proteins as the target antigen have been developed or licensed yet for human use. Intriguingly, the genome of chimeric flavivirus vaccines (JE-CVax/Imojev or CYD-TDV/Dengvaxia) consists of sequences of antigenically distinct flaviviruses (respectively, JEV and YFV-17D or DENV and YFV-17D) and may therefore exert some dual protective activities. Here we demonstrate that vaccination of mice with a construct similar to JE-CVax/Imojev provides rapidly complete protection against a massively lethal YFV challenge, with a single dose being sufficient for full efficacy. Moreover, we show that this protection is, notwithstanding its unexpected potency, mediated not by nAbs but by multiple complementary and vigorous responses directed against the NS proteins of YFV-17D.

## RESULTS

### JE-CVax provides full dual protection against lethal JEV and YFV challenge in mice.

**FIG 1:**
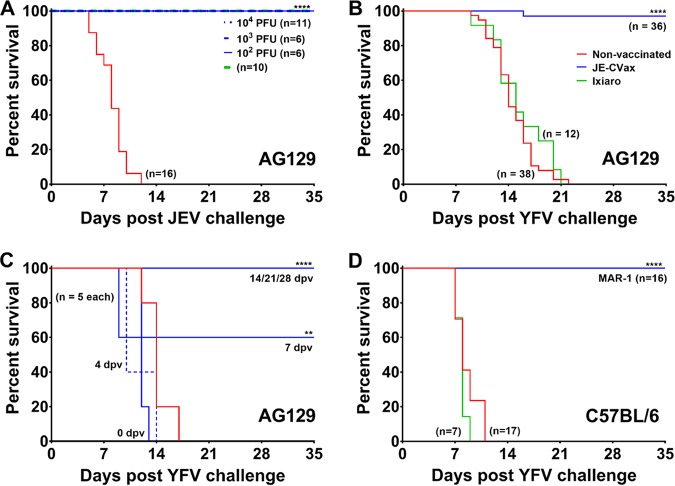
*In vivo* evaluation of JE-CVax-mediated dual protection against lethal JEV SA14-14-2 and YFV-17D challenge. (A to D) AG129 and C57BL/6 mice were first vaccinated via the i.p. route with either 10^4^ PFU of JE-CVax (blue), 1/6th of a human dose of Ixiaro (green), or assay medium as a negative control (red). Animals vaccinated with Ixiaro were boosted with another 1/6th of a human dose of Ixiaro 14 dpv. In order to facilitate vaccine virus replication (28), wild-type C57BL/6 mice receiving JE-CVax vaccination were treated with MAR1-5A3 antibody. AG129 mice were i.p. challenged with 10^3^ of PFU JEV SA14-14-2 at 28 dpv (A) or with 10^3^ PFU of YFV-17D at 28 dpv (B) or at 0, 4, 7, 14, 21, and 28 dpv (C). C57BL/6 mice were i.c. challenged with 10^4^ PFU of YFV-17D at 28 dpv (D). Animals were observed for 5 weeks after challenge and were euthanized when humane endpoints were reached. The data represent cumulative results of at least two independent experiments. Log rank (Mantel-Cox) survival analysis test was performed for statistical significance. **, *P* ≤ 0.01; ****, *P* ≤ 0.001 compared to the nonvaccinated group.

JE-CVax is a c-LAV that consists of the YFV-17D genome, of which the prM and E genes have been replaced by the corresponding sequences of JEV SA14-14-2. AG129 mice were vaccinated with either 103 or 104 PFU of JE-CVax and 28 days later challenged with 103 PFU (equivalent of 1,000 50% lethal doses [LD50]) of YFV. This resulted in, respectively, 80 or 100% survival, while YFV infection was uniformly lethal in all nonvaccinated controls (see Fig. S1A in the supplemental material). Therefore, throughout the rest of the study, animals were vaccinated and challenged with 10^4^ PFU of JE-CVax (full survival in vaccinated mice) and 10^3^ PFU of YFV (full mortality in nonvaccinated mice). JE-CVax was originally developed as a JEV vaccine. As expected, unlike nonvaccinated animals (*n* = 16), all AG129 mice vaccinated with either JE-CVax (10^2^, 10^3^, or 10^4^ PFU; *n* ≤ 6) or the inactivated JEV vaccine Ixiaro (*n* = 10; 2 times at 1 μg: twice 1/6th human dose) (25) were completely protected (*P* > 0.0001) against lethal JEV challenge (Fig. 1A). Remarkably, vaccination with JE-CVax resulted also in 97% survival (*n* = 35/36) against a massively lethal YFV challenge (Fig. 1B). All placebo-vaccinated (*n* = 38) and Ixiaro-vaccinated (*n* = 12) animals had to be euthanized for humane reasons (mean days to euthanasia [MDE], 14.6 ± 2.8 days and 15.4 ± 3.5, *P* > 0.0001). Importantly, JE-CVax also conferred similarly vigorous protection against YFV in C57BL/6 wild-type (wt) mice (*n* = 16) against intracranial (i.c.) challenge with 10^4^ PFU of YFV (Fig. 1D). In AG129 mice, a benefit (60% survival) could already be observed 7 days postvaccination (dpv). At 14 dpv or later, all animals were fully protected against lethal challenge (Fig. 1C). To establish that JE-CVax-mediated protection against YFV is specific and not the result of some residual cross-reactivity as previously observed for certain flaviviruses in mice (26), we challenged age-matched nonvaccinated (*n* = 7) or JE-CVax-vaccinated and YFV-17D-challenged (*n* = 6) AG129 mice with 10^4^ PFU of the more distantly related ZIKV (strain MR766). No protective activity was observed (MDE for nonvaccinated and vaccinated mice, 23.5 ± 5.4 and 17.4 ± 8.8 days; *P* = 0.4831) (Fig. S1B). Thus, a single-dose immunization with JE-CVax provides fast (≤14 dpv) and virus-specific protection against lethal YFV exposure in mice.

10.1128/mBio.02494-19.1 FIG S1 *In vivo* characterization of protective efficacy of JE-CVax vaccination against lethal YFV-17D and ZIKV-MR766 challenge in AG129 mice. (A) AG129 mice (*n* = 5) were vaccinated i.p. with 10^3^ to 10^4^ PFU of JE-CVax (blue) and at 28 dpv challenged i.p. with 10^3^ PFU of YFV-17D. (B) AG129 mice (*n* = 6) were first vaccinated i.p. with 10^4^ PFU JE-CVax and at 28 dpv challenged i.p. with 10^3^ PFU of YFV-17D (blue). At 28 days post-YFV-17D challenge, animals were challenged a second time with 10^4^ PFU of ZIKV-MR766 and observed for mortality for the following 5 weeks. Age-matched nonvaccinated (red) animals were challenged with 10^3^ PFU of YFV-17D (*n *= 5 [A]) or 10^4^ PFU of ZIKA-MR766 (*n *= 7 [B]) as a control. Log rank (Mantel-Cox) survival analysis test was performed for statistical significance. **, *P* ≤ 0.01 compared to the nonvaccinated group. Download FIG S1, JPG file, 0.2 MB. Copyright © 2020 Mishra et al. 2020 Mishra et al. This content is distributed under the terms of the Creative Commons Attribution 4.0 International license.

### JE-CVax mediates protection against YFV without involvement of nAbs.

**FIG 2:**
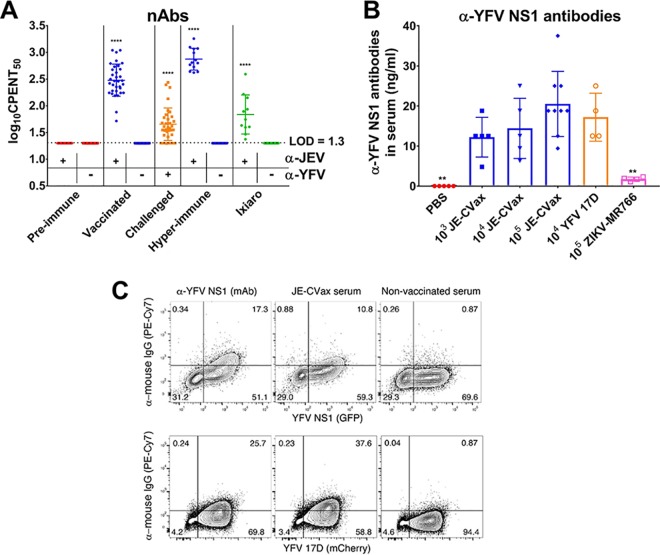
Serological analysis of serum of JE-CVax-vaccinated and YFV-17D- or ZIKV-MR766-challenged animals. (A) Detection of nAbs against JEV and YFV. CPE neutralization tests (CPENT) for JE-CVax (circles) and YFV-17D (squares) were performed on sera day 0 prior to vaccination (preimmune, red), day 28 after vaccination (blue), and after challenge (study endpoint, orange) for samples of JE-CVax-vaccinated AG129 mice, of JE-CVax-vaccinated mice after subsequent YFV-17D-challenge (*n* = 34), of mice hyperimmunized with JE-CVax (*n* = 13; first bleed 2 weeks post last booster immunization, blue), and of mice vaccinated with Ixiaro (green). Limit of detection (LOD) for virus neutralization was log_10_ 20 (1.3). Data are presented as log_10_ CPENT_50_ (mean ± SD). The data presented are from ≥3 independent experiments. Statistical significance was determined using one-way ANOVA. ****, *P* ≤ 0.0001 for mean log_10_ CPENT_50_ titers against JEV or YFV compared to mean log_10_ CPENT_50_ titers before JE-CVax vaccination and before YFV-17D-challenge, respectively. (B) Quantitation of anti-YFV NS1 binding antibodies by direct ELISA. Serum from naive, nonvaccinated mice (red) or mice that had been vaccinated with 10^3^ to 10^5^ of PFU JE-CVax (blue) or that had been infected with 10^4^ PFU of YFV-17D (orange) or with 10^5^ PFU of ZIKV-MR766 (pink) were collected either 28 days postimmunization or when euthanized at the humane endpoint (*n* ≥ 5). The data are means of two independent analyses. Statistical significance was determined using one-way ANOVA. **, *P* ≤ 0.01 compared to YFV-17D. (C) Binding of serum antibodies to NS1-expressing cells. HEK 293 cells were transfected with a plasmid expressing YFV-17D NS1 as a transcriptional fusion to GFP (top) or infected with the YFV-17D-mCherry reporter virus (bottom). Either 48 h after transfection or 72 h after infection, cells were stained with the anti-YFV NS1-specific MAb 1A5 (left), with serum from mice that were vaccinated with JE-CVax (center), or with serum from naive, nonvaccinated mice (right). Graphs show flow cytometric analysis of GFP or mCherry fluorescence and visualization of anti-YFV NS1 antibody binding using a PE-Cy7-conjugated goat anti-mouse IgG secondary antibody. The fraction of NS1-positive cells (GFP or mCherry) stained by MAb 1A5 or serum of JE-CVax-immunized mice (anti-mouse IgG) is given as a percentage in the upper right quadrant. Data from one representative experiment out of four independent experiments are shown.

To explore whether humoral immunity is involved in JE-CVax-mediated protection against YFV, serum of AG129 mice (i) at day 0 (prevaccinated), (ii) infected with YFV-17D before euthanasia (terminal serum), (iii) vaccinated with JE-CVax (day 28; postvaccinated), or (iv) vaccinated with JE-CVax and challenged with YFV-17D (day 56; postchallenge) was analyzed for total binding antibodies and nAb. All animals vaccinated with JE-CVax or Ixiaro seroconverted to JEV positivity. Sera of nonvaccinated animals that had been infected with YFV-17D showed only some residual reactivity for JEV (as detected by indirect immune fluorescence assay [IIFA] [Fig. S2]). In contrast, nAbs against JEV were exclusively detected in serum samples of JE-CVax- or Ixiaro-vaccinated animals (50% neutralizing antibody titer as determined using CPE-based virus neutralization tests [log_10_ CPENT_50_], 2.48 ± 0.29 or 1.86 ± 0.36, respectively) (Fig. 2A). Only when JE-CVax-vaccinated mice were challenged at a later stage with YFV-17D were nAbs against the latter virus raised (log_10_ CPENT_50_, 1.66 ± 0.30; determined 28 days after YFV exposure). Also in serum of JE-CVax- or Ixiaro-vaccinated C57BL/6 mice, only nAbs against JEV (log_10_ CPENT_50_, 1.66 ± 0.12 or 1.61 ± 0.09, respectively) were detectable. However, all the C57BL/6 mice intraperitoneally (i.p.) vaccinated with 10^4^ PFU YFV-17D in the presence (*n* = 8) or absence of MAR1 (*n* = 9) developed nAbs against YFV (log_10_ CPENT_50_, 2.11 ± 0.25 or 1.78 ± 0.29, respectively) and survived lethal i.c. challenge of YFV. Thus, neither JE-CVax nor Ixiaro induces YFV-specific nAbs in mice.

10.1128/mBio.02494-19.2 FIG S2 Detection of cross-reactive antibodies against JEV and YFV in sera of YFV-17D-infected and JE-CVax-vaccinated mice. AG129 mice were prebled before either infection with 10^3^ PFU of YFV-17D or vaccination with 10^4^ PFU of JE-CVax/Ixiaro and bled again either at the onset of sickness (YFV-17D) or at 28 dpv (JE-CVax). Preserum (A), serum of YFV-17D infected mice (B), serum of JE-CVax-vaccinated mice (C), and serum of Ixiaro-vaccinated mice (D) were analyzed by both JEV (top) and YFV (bottom) indirect immunofluorescence assay (IIFA; Euroimmun) at a magnification of ×20. Download FIG S2, JPG file, 0.4 MB. Copyright © 2020 Mishra et al. 2020 Mishra et al. This content is distributed under the terms of the Creative Commons Attribution 4.0 International license.

### JE-CVax and YFV-17D induce comparable levels of anti-NS1 YFV antibodies.

From the IIFA analysis (Fig. S2), it is obvious that sera from JE-CVax-vaccinated mice contain cross-reactive but nonneutralizing Abs against YFV-17D (Fig. 2A). These non-nAbs may possibly be attributed to NS1, a strong B cell antigen. To assess the presence of anti-YFV NS1 antibodies in JE-CVax-vaccinated AG129 mice, a direct enzyme-linked immunosorbent assay (ELISA) was performed on sera from mice vaccinated with 10^3^ to 10^5^ PFU of JE-CVax or mice infected with 10^4^ PFU of YFV-17D or 10^5^ of PFU ZIKV-MR766. Serum was obtained either at the onset of disease (YFV-17D, 10^5^ PFU of JE-CVax and ZIKV) or at 28 dpv. Levels of anti-YFV NS1 antibodies in the different JE-CVax-vaccinated groups were statistically not different (*P* > 0.05) from those in the YFV-17D-infected groups (Fig. 2B). Moreover, very low cross-reactivity was noted for samples from ZIKV-infected mice. These findings were also confirmed by flow cytometry analysis (Fig. 2C). Serum antibodies from JE-CVax-vaccinated mice bound to cells that overexpress YFV NS1 as well as to cells that had been infected with YFV-17D. In fact, serum from JE-CVax-vaccinated mice resulted in staining comparable to that obtained when monoclonal antibody (MAb) 1A5, specifically directed against YFV NS1, was used (27).

### JE-CVax induces YFV-specific antibodies mediating ADCC.

**FIG 3:**
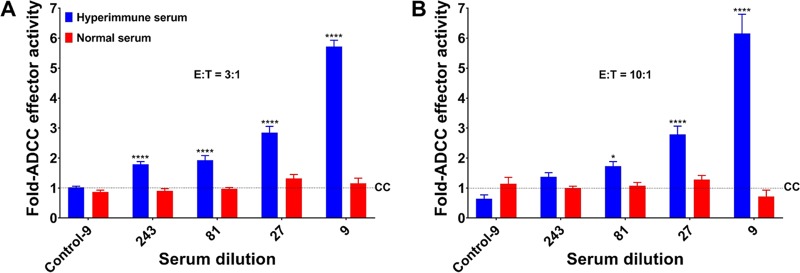
Role of antibody-dependent cell-mediated cytotoxicity (ADCC) conferred by JE-CVax hyperimmune serum in the protection against YFV. JE-CVax hyperimmune serum was tested for its ability to mediate ADCC activity compared to serum of nonvaccinated mice (normal serum) at 3:1 (A) and 10:1 (B) effector (E)-to-target (T) cell ratios. Experiments were conducted twice, each in triplicate, and data are presented as means ± SEMs for fold changes compared to control (CC) (i.e., mean reporter signal plus three SDs from E:T in the absence of hyperimmune serum). Values from noninfected target cells incubated with E in the presence of either hyperimmune serum or normal serum at highest antibody concentrations (dilution 1:9) are indicated as Control-9. Statistical significance was determined using two-way ANOVA. * and ****, *P* ≤ 0.05 and 0.0001 compared to normal serum.

To determine the potential mechanism that non-nAbs elicit for protection against YFV, an antibody-dependent cellular cytotoxicity (ADCC) reporter bioassay was carried out using HEK293T cells infected with YFV-17D (expressing mCherry) (28) as target cells and murine Fcγ receptor IIIA (FcγRIIIa)-expressing Jurkat reporter cells as effector cells (29). Hyperimmune mouse serum from JE-CVax-vaccinated AG129 mice induced clear ADCC responses and in a dose-dependent manner, whereas serum from nonvaccinated mice failed to do so (Fig. 3 and Fig. S3A and B).

10.1128/mBio.02494-19.3 FIG S3 Effect of JE-CVax hyperimmune serum in antibody-dependent cellular cytotoxicity (ADCC). JE-CVax hyperimmune serum (circles) was tested for its ability to mediate ADCC activity in comparison to serum of nonvaccinated mice (squares) at 3:1 (A) and 10:1 (B) effector (E)-to-target (T) ratios. Data are from experiments conducted twice, each in triplicate, and presented as means ± SEMs. The average of relative light unit signal plus three standard deviations from E:T ratios in the absence of hyperimmune serum was considered the background signal (CC). Statistical significance was determined using two-way ANOVA. *, **, ***, and ****, *P* ≤ 0.05, 0.01, 0.001, and 0.0001 compared to normal serum. Download FIG S3, JPG file, 0.2 MB. Copyright © 2020 Mishra et al. 2020 Mishra et al. This content is distributed under the terms of the Creative Commons Attribution 4.0 International license.

### JE-CVax induces polyfunctional T cell responses against both YFV and JEV antigens.

**FIG 4:**
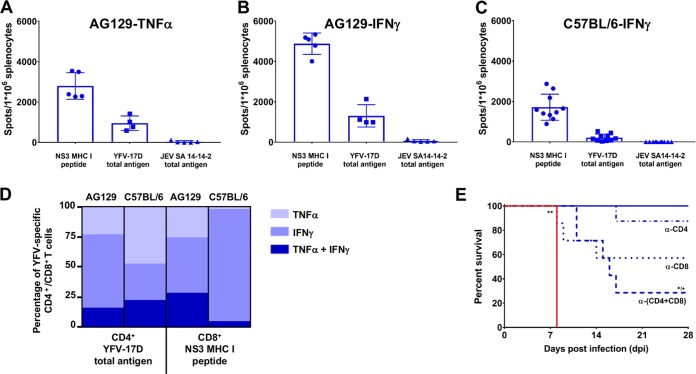
Detection of protective T cell responses directed against YFV. (A to C) ELISpot assay data showing TNF-α (A) IFN-γ (B and C) and production by splenocytes of AG129 mice (*n* = 5; A and B) or C57BL/6 (*n* = 10; C) at 18 and 4 weeks, respectively, after vaccination with 10^4^ PFU of JE-CVax, following 16 h *ex vivo* restimulation with either an MHC class I-restricted peptide derived from YFV-17D NS3 (32) or the lysate of YFV-17D- or JEV SA14-14-2-infected Vero E6 cells. Stimulation using lysate of noninfected Vero E6 cells served as a negative control. The data are derived from two independent experiments. Spot counts were normalized by subtraction of the number of spots in corresponding wells stimulated with uninfected Vero E6 cell lysate. (D) Cytokine expression profile of YFV-specific T cells. Shown are IFN-γ and TNF-α production profiles of YFV-specific CD4^+^ and CD8^+^ T cells from JE-CVax-vaccinated AG129 and C57BL/6 mice 18 and 4 weeks, respectively, postvaccination, as determined by intracellular cytokine staining. Mouse splenocytes were stimulated 16 h *ex vivo* with either an MHC class I-restricted NS3 peptide, cell lysate of YFV-17D-infected Vero E6 cells, or lysate of uninfected Vero E6 cells. The data are derived from two independent experiments and normalized by subtraction of number of cytokine-secreting T cells in corresponding samples in which uninfected Vero E6 cell lysate was used as recall antigen. (E) T cell-mediated *in vivo* protection against YFV. Loss of protection resulting from antibody-mediated T cell depletion (30, 52) suggests a direct functional involvement of CD4^+^ and CD8^+^ T cells in JE-CVax-mediated immunity against YFV in C57BL/6 mice (*n* ≥ 7) that had been vaccinated with 10^4^ PFU of JE-CVax and subsequently challenged intracranially with 10^4^ PFU of YFV-17D. Depletions were performed by administration of 0.5 mg of anti-mouse CD4 and/or anti-mouse CD8 antibodies i.p. on day −2 and day 0 each prior to YFV challenge. Log rank (Mantel-Cox) survival analysis test was performed for statistical significance. * and **, *P* ≤ 0.05 and 0.01 compared to vaccinated group (*n* = 5); ^+^, *P* ≤ 0.05 compared to CD4-depleted group (*n* = 8).

To assess whether also cellular immune response against YFV may contribute to the protective activity, enzyme-linked immunosorbent spot (ELISpot) assays (tumor necrosis factor alpha [TNF-α] and/or gamma interferon [IFN-γ]) and intracellular staining of cytokines (TNF-α and IFN-γ) were performed on total splenocytes obtained from AG129 mice (*n* = 5) and C57BL/6 mice (*n* = 10) 18 and 4 weeks after JE-CVax immunization, respectively. Unlike the case with splenocytes of nonvaccinated mice (Fig. S6E to H), robust and specific IFN-γ and/or TNF-α production was observed from splenocytes of either mouse strain vaccinated with JE-CVax (Fig. 4A to C and Fig. S4) after recall with a major histocompatibility complex (MHC) class I-restricted peptide derived from YFV-17D NS3 or YFV-17D total cellular antigen. In line, flow cytometric analysis revealed robust and YFV-specific intracellular cytokine production in CD4^+^ and CD8^+^ T cells from spleens of JE-CVax-vaccinated AG129 and C57BL/6 mice when stimulated *ex vivo* with the YFV NS3 peptide or YFV-17D total cellular antigen (Fig. 4D and Fig. S4 to S6). Overall, JE-CVax vaccination induced specific long-lasting T cell responses against YFV, and cellular immunity against YFV was more vigorous than that elicited against JEV (Fig. 4A to C and Fig. S4 to S6). This observation is consistent with YFV NS proteins serving as more immunogenic T cell antigens than prM/E of JEV (30, 31).

10.1128/mBio.02494-19.4 FIG S4 T cell responses directed against YFV or JEV antigens in ELISpot assay. Representative wells of ELISpot assays showing TNF-α (A) and IFN-γ (B and C) production by splenocytes of AG129 mice (A and B) and C57BL/6 mice (C) at 18 and 4 weeks, respectively, after vaccination with 10^4^ PFU JE-CVax, following 16 h of *ex vivo* restimulation with an MHC class I-restricted peptide derived from YFV NS3^32^ or the lysate of YFV-17D- or JEV SA14-14-2-infected Vero E6 cells. Stimulation using lysate of noninfected Vero E6 cells served as a negative control. Download FIG S4, JPG file, 0.3 MB. Copyright © 2020 Mishra et al. 2020 Mishra et al. This content is distributed under the terms of the Creative Commons Attribution 4.0 International license.

10.1128/mBio.02494-19.5 FIG S5 Detection of T cell responses in mice by intracellular staining for TNF-α and IFN-γ using flow cytometry. Shown are results from flow cytometric analysis for intracellular TNF-α (A, B, E, and F) and IFN-γ (C, D, G, and H) production by CD4^+^ (A, C, E, and G) or CD8^+^ (B, D, F, and H) T cells from vaccinated AG129 mice (A to D) and C57BL/6 mice (E to H) following stimulation with either NS3 MHC class I peptide or cell lysate of YFV-17D or JEV SA14-14-2-infected Vero E6 cells. The percentage of total CD4^+^ or CD8^+^ TNF-α- or IFN-γ-secreting T cells analyzed in flow cytometric analysis in AG129 mice (*n* = 5) and C57BL/6 mice (*n* = 10) was determined. The tables in panels I and J represent *P* values between cytokine-secreting populations of antigen- versus noninfected Vero E6 cell-stimulated samples (statistical significance; paired *t* test) in flow cytometric analysis for splenocytes from AG129 and C57BL/6 mice, respectively. The data were compiled from two independent experiments, and dotted lines represent average background in control samples collected from non-vaccinated animals. Download FIG S5, JPG file, 0.5 MB. Copyright © 2020 Mishra et al. 2020 Mishra et al. This content is distributed under the terms of the Creative Commons Attribution 4.0 International license.

10.1128/mBio.02494-19.6 FIG S6 Detection of T cell responses through intracellular cytokine staining in vaccinated (A to D) AG129 and C57BL/6 mice versus nonvaccinated baseline controls (E to H). Representative depiction of flow cytometric analysis for intracellular TNF-α and IFN-γ production by CD4^+^ (gated CD3^+^ CD8^–^) (A and C) or CD8^+^ T cells (gated CD3^+^ CD8^+^) (B and D) from JE-CVax-vaccinated (A and B) AG129 mice, 18 weeks postvaccination and (C and D) C57BL/6 mice, 4 weeks postvaccination, following 16h *ex vivo* stimulation with lysate of YFV-17D- or JEV SA14-14-2-infected Vero E6 cells, an MHC class I-restricted NS3 peptide, or uninfected Vero E6 cells. Results of similar analyses of nonvaccinated AG129 (E and F) and C57BL/6 mice (G and H) are shown. Download FIG S6, JPG file, 0.7 MB. Copyright © 2020 Mishra et al. 2020 Mishra et al. This content is distributed under the terms of the Creative Commons Attribution 4.0 International license.

### Both CD4^+^ and CD8^+^ T cells contribute to JE-CVax-mediated protection against YFV.

To determine whether YFV-specific T cell responses directly contribute to the JE-CVax-mediated protection against YFV, T cell depletion experiments were performed with C57BL/6 mice (32). Animals at 5 weeks postvaccination were administered with anti-mouse CD4 and/or anti-mouse CD8a T cell-depleting antibodies twice, i.e., 2 days before and immediately prior to YFV challenge. Unlike for vaccinated (but not further treated) animals (that were included as immunization controls [*n* = 5] and that survived an intracranial YFV challenge), in vaccinated but antibody-treated mice the previously observed full protection against YFV was partially lost by targeting CD4^+^ (*n* = 1/8), CD8^+^ (*n* = 3/7), and CD4^+^ CD8^+^ (*n* = 5/7) T cells for depletion (Fig. 4E). All nonvaccinated animals (*n* = 5; *P* = 0.0027) succumbed to YFV challenge as before (Fig. 1D). The mortality resulting from T cell depletion, especially the increased mortality observed in the doubly depleted animal group, suggests that in the absence of nAb both CD4^+^ and CD8^+^ T cells contribute to the JE-CVax-mediated protection against YFV in C57BL/6 mice.

### CTL epitopes within the YFV-17D NS proteins have higher binding affinity toward human MHC class I molecules than those of mice.

Cytotoxic T lymphocyte (CTL) epitopes and their binding affinities toward respective MHC class I molecules are different in different species. *In silico* analysis of YFV-17D NS proteins was performed to compare the probable CTL epitopes and their binding efficiencies, using an algorithm that takes peptide processing, presentation, and MHC-peptide complex stability into account. Data presented in Fig. S10 indicate that as a predictor of immunogenicity, peptides of YFV-17D NS proteins have higher binding affinity to human MHC than to mouse MHC.

## DISCUSSION

Neutralizing antibodies against the E protein are generally considered a primary correlate of protection against flaviviruses (5, 15). However, some preclinical studies suggested that several NS proteins when used as immunogens for vaccination alone or in combination could induce some degree of protection against viral challenge in mice and nonhuman primates (17–20). For instance, a recombinant vaccinia virus or replication-deficient adenoviral vectors expressing YFV-17D NS1, NS2a, and NS2b together or NS3 alone, respectively, resulted in some partial protective immunity against a lethal challenge of YFV-17D in mice (17, 23). However, full survival could never be achieved and reached maximally 60 to 80%, versus 100% for YFV-17D-vaccinated controls. Similarly, 80% protection against challenge with the African YFV strain Dakar 1279 was observed in monkeys following repeated immunization with purified NS1 as the sole vaccine antigen, and protection correlated with the levels of non-nAbs against NS1 (19). Conversely, vaccination of mice with E protein alone (or in combination with NS1 or NS3) resulted in complete protection (23, 24).

Like Imojev (commercially available live attenuated JE vaccine), our live attenuated JE-CVax vaccine is a chimeric flavivirus that consists of the genome of YFV-17D from which the prM/E genes have been replaced by those of JEV SA14-14-2, and hence, it is nearly identical to Imojev (i.e., a retroengineered genetic copy) but not formally the same live attenuated virus. We therefore used JE-CVax to assess whether it can offer, besides protection against JEV, protection against YFV challenge. Since AG129 mice are highly susceptible to lethal JEV SA14-14-2 and YFV-17D infection (Fig. S7) (28, 33), we used these two vaccine strains as established surrogates for wt JEV (33) and wt YFV (34, 35), respectively (demanding lower biosafety containment for handling). A single dose of JE-CVax provided, in addition to the expected protection against JEV challenge, nearly complete protection (35, 36) against a massive (1,000 LD_50_] challenge with YFV (Fig. 1B). The protective activity against YFV was raised fast, and a survival benefit could be observed already within 7 days after vaccination (Fig. 1C). These observations were further corroborated by the fact that vaccination of immunocompetent C57BL/6 mice with a single dose of JE-CVax provided complete protection (21/21) against lethal intracranial challenge (17, 23) with YFV (Fig. 1D and Fig. 4E). This activity of JE-CVax was YFV specific, as AG129 mice that had been vaccinated with Ixiaro (the inactivated JEV vaccine) were not protected against lethal YFV challenge. Furthermore, mice that had been vaccinated with JE-CVax and that later survived YFV challenge did not survive a subsequent lethal challenge with the ZIKV (Fig. S1).

10.1128/mBio.02494-19.7 FIG S7 *In vivo* infectivity of YFV-17D and JEV SA14-14-2 in AG129 mice. AG129 mice were inoculated via the i.p. route with different doses of YFV-17D (10^0^ PFU [orange solid line; *n* = 16], 10^1^ PFU [orange dashed line; *n* = 3], or 10^2^ PFU [orange dotted line; *n* = 3]) (A) or JEV SA14-14-2 (10^0^ PFU [purple solid line], 10^1^ PFU [purple dashed line], or 10^2^ PFU [purple dotted line]; *n* =3) (B). Animals were monitored over a period of 5 weeks and were euthanized when humane endpoints were reached. Download FIG S7, JPG file, 0.2 MB. Copyright © 2020 Mishra et al. 2020 Mishra et al. This content is distributed under the terms of the Creative Commons Attribution 4.0 International license.

Others have shown in mice that cross-reactive antibodies (together with cross-reactive T cells) may confer partial protection against flaviviruses from different serocomplexes, as demonstrated for JEV (both Vero cell-derived JEV-P3 strain-based inactivated vaccine and JEV SA14-14-2)-vaccinated mice challenged with DENV (26). Likewise, a chimeric Japanese encephalitis/dengue virus 2 experimental vaccine, ChinDENV (originally designed to induce immunity against DENV-2 prM/E), was shown to protect against both JEV and DENV-2 challenge in mice (31, 36). The protection observed could, however, still largely be explained by the induction of considerable levels of E-specific partially cross-reactive nAbs that neutralized both DENV and JEV. Additionally, the study (31) demonstrated that vaccination also induces JEV antigen-specific T cell responses (T cells producing IFN-γ and interleukin 2 [IL-2] following stimulation with JEV antigen), suggesting a possible contribution of the cellular immunity in the defense against JEV challenge in ChinDENV-immunized mice. Another study, in which AG129 and IFNAR mice were used, reported that during heterotypic dengue virus infection, CD8^+^ T cells provide some degree of protection in the absence of detectable levels of nAbs (37). However, in this case a mild (sublethal) DENV-4 infection was used for priming rather than a true vaccine, and despite this priming, only limited (partial) and short-term protection was observed against DENV-2. Moreover, the E proteins of DENV-2 and DENV-4 share high (∼64%) sequence homology, including conserved T cell epitopes (38), and therefore, the contribution of the E protein to protective T cell responses could obviously not be distinguished from what DENV NS proteins may contribute. In contrast, although we also observed some residual cross-reactivity for YFV in serum samples of JE-CVax-vaccinated mice (binding antibody in IIFA [Fig. S2]), JE-CVax failed to induce any detectable nAb titers against YFV even following repeated boosting (Fig. 2A). This finding is in full accordance with the absence of cross-nAbs in mice, monkeys, and humans after JE-CVax or YFV-17D vaccination (39–41). Therefore, cross-reactivity of serum from JE-CVax-vaccinated mice could be attributed to (i) induction of E-based broad flavivirus cross-reactive non-nAbs resulting from flavivirus infection/vaccination (26, 42) and (ii) reactivity against YFV-NS1 that is expressed within and on the surface of infected cells and is target of specific binding but non-nAbs (20, 43). In fact, we demonstrate equivalent levels of anti-YFV-NS1 antibodies in JE-CVax-vaccinated and YFV-17D-infected mice (Fig. 2B and C).

Although we show that JE-CVax immunization resulted in complete protection against YFV-induced disease, there were variations in the actual levels of anti-YFV nAbs after YFV challenge. In some animals, no nAbs were detected against YFV after YFV-17D-challenge. This lack of YFV nAb indicates that JE-CVax possibly conferred sterilizing immunity in these mice. Since such protection cannot be explained by nAb against the YFV-prM/E, it may be accredited to non-nAbs (42–44) and/or adaptive cellular immunity (16, 30, 32, 45). Some correlation between anti-NS1 antibody levels and the dose of JE-CVax needed to cause protection was observed (Fig. 2B and C and Fig. S1A), and serum from JE-CVax-vaccinated mice was found to induce an ADCC response against YFV-17D (Fig. 3 and Fig. S3). In addition, in our model both CD4^+^ and CD8^+^ T cells seem (in a likely association with anti-NS1 antibodies) to be involved in JE-CVax-mediated protection against YFV (Fig. 4E). Previously, only humoral immunity and CD4^+^ T (but not CD8^+^ T) cells have been implied to be sufficient and required for protection against YFV (16), with a strong emphasis on nAb as a historically established immunological correlate of protection for YFV (5) and for flaviviruses (such as JEV) in general (1, 15, 39). nAbs possibly block viral spread, whereas cellular immunity efficiently eliminates intracellular viruses either directly or targeted by non-nAbs toward infected cells in an Fc-dependent manner (for example, via ADCC toward cells expressing YFV NS1 on their surfaces) (18, 21, 43, 44). Indeed, YF-induced CD8^+^ T cells have been shown to act as a “backup defense” system in the absence of nAbs and to participate in viral clearance in particular from the central nervous system (CNS) in mice (23, 32). Moreover, strong CD8^+^ T cell responses are also detected in the response to human vaccines (30). As we show here, immunization of AG129 and C57BL/6 mice with JE-CVax elicited protective polyfunctional YFV antigen-specific CD4^+^ and CD8^+^ T cell responses (expressing the Th1-type cytokines TNF-α and IFN-γ), which is in line with a previous study with a chimeric Japanese encephalitis/dengue virus 2 vaccine (31). Previously, clinical studies with humans also demonstrated that vaccination with CYD-TDV/Dengvaxia elicits cell-mediated immunity directed against YFV nonstructural proteins (46, 47). Our comparative *in silico* analysis indicated that the YFV-17D backbone is more immunogenic in humans than in mice and provides a hint that CTL responses should be at least as effective in humans as in mice. Likewise, several HLA-binding YFV-17D peptides thus predicted have recently also been confirmed experimentally using human-derived T cells and vaccinated human volunteers (48–51) (Fig. S10). Collectively, our data suggest that JE-CVax-mediated vigorous protection against lethal YFV challenge depends on the combined effects of several effector principles, including both the humoral and cellular immune responses, yet definitely other than nAbs.

To assess the efficacy of JE-CVax, we employed mice as the *in vivo* model and the YFV-17D vaccine strain (16, 17, 23, 44, 52) as the challenge virus. This experimental setup implies some constraints. Generally, mice are not susceptible to human flavivirus infection (53) and the YFV-17D is not virulent in humans (5). To overcome these limitations, we made use of two established, complementary and stringent mouse infection models that are accepted surrogates for testing of flavivirus vaccines (11); (i) immunocompromised mice (AG129 mice [28, 34, 35]) that develop fatal neurotropic infection when challenged peripherally with YFV-17D, in particular when inoculated with a highly lethal (1,000 LD_50_) challenge virus dose (Fig. S7) (28), and (ii) immunocompetent wt mice (C57BL/6 [16, 17, 23] that can be challenged i.c. with YFV-17D. YFV-17D was originally developed by adapting a viscerotropic clinical isolate (YFV-Asibi) to replication in mouse brains (“fixed to mouse brain”) (54). This vaccine virus can hence be considered a genuine mouse-adapted neurotropic and neurovirulent YFV strain. For this reason, it could also be considered in this study as the challenge virus. Besides experimental convenience (YFV-17D does not require biosafety level 3 [BSL3] containment), YFV-17D is a widely accepted, i.e., best-characterized and hence most widely used, challenge strain in mouse models. Classical i.c. inoculation of YFV-17D consistently causes fatal disease in mice (52) that cannot be distinguished from that induced by wt-YFV strains (44, 52). Also, because JE-CVax expresses the prM/E protein of JEV, which belongs to a serocomplex other than YFV, vaccination and subsequent challenge with YFV-17D compares to a certain extent to a heterotypic flavivirus vaccination challenge (as described by others [37]), however, with a markedly more pronounced vaccine efficacy. Therefore, in conclusion, similar mechanisms should hold when using wild-type YFV as the challenge virus (44, 52). Obviously, before proceeding to clinical use, this principle should be confirmed in JE-Cvax-vaccinated nonhuman primates, demonstrating protection from subsequent challenge with virulent wt YFV strains.

Live attenuated JE SA14-14-2 and inactivated Vero cell vaccine account for the biggest use by volume, while Imojev is licensed and used in only a few Asian countries. It is evident from the literature that Imojev remains safe and immunogenic in individuals with a known history of JE vaccination (55). However, further studies are required to address whether Imojev will also induce protective immunity against YFV in those that have already been vaccinated with one of the other JE vaccines. In any case, formal demonstration of vaccine efficacy in humans will be challenging considering that YFV-specific nAbs that are established as a correlate of protection for YFV-17D are absent (39–41), and hence protection against lethal infection may instead need to be shown in nonhuman primate vaccine challenge models. Previous safety and immunogenicity studies of chimeric live attenuated viruses (JE-CVax and ChimeriVax-DEN2) in humans indicated that preexisting immunity to the parental YFV-17D vaccine virus does not interfere with immunization but rather induces long-lasting cross-neutralizing antibody responses (39, 56, 57). Importantly, if our data on the dual protection conferred by c-LAV in mice could be translated to other species, including humans, this would imply that the JE vaccine JE-CVax (and likewise Imojev) may provide dual protection, i.e., against both JEV and YFV. A dual protective effect may be of particular relevance in case YFV may one day—as is suspected (8, 9)—cause outbreaks in (sub)tropical Asia. Given the capacity problems with the production of the current YFV vaccine, having another licensed vaccine (i.e., JE-CVax/Imojev) available as an alternative means to protect against YFV may at such time help to contain an outbreak. In addition, those already vaccinated with JE-CVax may be expected to be protected against YFV. A similar principle may apply to other chimeric flavivirus vaccines that consist of a YFV-17D backbone (such as CYD-TDV/Dengvaxia) (12) and others under development (such as our recently proposed chimeric YFV-17D/ZIKV vaccine candidate) (28). Likewise, c-LAVs could be generated against DENV and other viruses that may cause ADE using the backbone of the parent virus (e.g., DENV) from which the prM/E genes have been replaced by that of antigenically more distantly related viruses or serotypes, in line with experimental evidence that the DENV NS1 protein can also serve as a protective antigen (81). Such an approach may avoid potentially harmful nAb responses. The same principle may apply to c-LAV for ZIKV using a ZIKV backbone (58) and prM/E sequences of another flavivirus that has not been shown to cause ADE.

To conclude, we demonstrate that JE-CVax efficiently and rapidly induces high cross-protective efficacy (∼100%) in mice against YFV challenge, even with an exceedingly aggressive challenge inoculum. The study provides evidence that c-LAV flavivirus vaccines may be developed solely based on NS proteins. Moreover, immunization with a chimeric flavivirus, whereby the prM/E genes of the backbone have been replaced by that of yet another flavivirus, may have a dual protective effect. A vaccine such as Imojev/JE-CVax may thus be suitable for off-label use, namely, for protection against YFV, which in this case is not mediated by nAbs.

## MATERIALS AND METHODS

### Cells and medium.

BHK-21J and Vero E6 cells used in this study were a generous gift from Peter Bredenbeek, Leiden University Medical Center (LUMC), the Netherlands. Cells were maintained in seeding medium containing MEM Rega-3 medium (Gibco, Belgium) supplemented with 10% fetal calf serum (FCS; Gibco), 2 mM glutamine (Gibco), and 0.75% sodium bicarbonate (Gibco). HEK 293 cells (human embryonic kidney 293 cells; ATCC CRL-1573) were cultured in Dulbecco modified Eagle medium (DMEM; Gibco) containing 10% FCS and 100 U/ml of penicillin-streptomycin solution (pen-strep; Gibco). Virus culture and cytopathic effect-based virus neutralization testing (CPENT) were performed in assay medium, which was the seeding medium supplemented with only 2% FCS. HEK 293 cells were transfected with YF-NS1-GFP using TransIT-LT1 transfection reagent (Mirus Bio LLC, Belgium), according to the manufacturer’s instructions. Infection of HEK 293 cells with YFV-17D-mCherry (see below) was performed in DMEM supplemented with 2% FCS and 100 U/ml of pen/strep solution. All cultures were maintained at 37 °C in an atmosphere of 5% CO_2_ and 95% to 99% humidity.

### Virus, vaccines, and antigens.

Stamaril (G5400) and Ixiaro (JEV16F290) were from Sanofi Pasteur (France) and Valneva (Austria), respectively. Stamaril was passaged two times in Vero E6 cells (YFV-17D-G5400P2) and stored at −80°C. YFV-17D-G5400P2 was used throughout the study to challenge mice and is referred to as YFV-17D. YFV-17D-based Japanese encephalitis c-LAV Imojev (Chimerivax-JE, JE-CVax) is not available in Europe and was, therefore, retroengineered according to publicly available sequence information (59) (patent number WO2006044857A2). To that end, a DNA fragment encoding the prM and E proteins of JEV vaccine strain SA14-14-2 was custom synthetized (IDT Integrated DNA Technologies, Haasrode, Belgium) and subcloned into the YFV-17D expression vector pShuttle-YFV-17D (59) (patent number WO2014174078 A1) of our plasmid-launched live attenuated vaccine (PLLAV)-YFV-17D platform using standard molecular biology techniques and thereby replacing the YFV-17D prM/E sequences. Two adaptive mutations in NS2A and NS4B genes and an additional Kas1 site at the end of the prM/E coding sequence (59) were introduced by site-directed mutagenesis. To generate JE-CVax virus, BHK-21J cells were transfected with PLLAV-JE-CVax using TransIT-LT1 transfection reagent, following the manufacturer’s instructions. Upon onset of cytopathic effect (CPE), JE-CVax virus was harvested, centrifuged at 4,000 rpm at 4°C for 10 min, aliquoted, and stored at −80°C. Similarly, the live attenuated Japanese encephalitis virus vaccine JEV SA14-14-2 (GenBank accession no. AF315119.1) was generated fully synthetically from overlapping DNA fragments (IDT Integrated DNA Technologies, Haasrode, Belgium), assembled by overlap extension PCR, and subsequent homologous recombination in yeast. The recombinant JE-CVax and JEV SA14-14-2 viruses were subsequently passaged on Vero E6 cells to generate virus stocks. As an alternative challenge virus, ZIKV strain MR766 was used (60). Virus titers were determined on BHK-21J cells by plaque assays (PFU per milliliter) and CPE-based assays (50% tissue culture infective doses [TCID_50_] per milliliter) as described below.

A YFV-17D reporter virus (YFV-17D-mCherry) was generated that expresses the red fluorescent protein mCherry as a translational fusion to the N terminus of YFV-17D C protein. In brief, using standard PCR techniques and homologous recombination in *Saccharomyces cerevisiae* (strain YPH500), a synthetic DNA fragment encoding codons 2 to 236 of mCherry (GenBank accession no. AY678264.1) was inserted in YFV-17D genome (61) immediately downstream of nucleotide (nt) position 181, flanked (i) at its 5′ terminus by a glycine-serine linker (BamHI site) and (ii) at the 3′ end by a Thosea asigna virus 2A peptide (EGRGSLLTCGDVEENPG/P) (62) followed by a repeat of codons 2 to 21 of the YFV-17D C gene, yet with an alternative codon usage to avoid RNA recombination during viral replication. YFV-17D-mCherry was rescued by transfection of the resulting PLLAV-YFV-17D-mCherry of BHK 21J as described previously (28). A full characterization of YFV-17D-mCherry is available upon request (M. A. Schmid, N. Mishra, S. Sharma, R. Boudewijns, J. Neyts, and K. Dallmeier, unpublished data).

Plasmid pCMV-YFV-17D NS1-IRES-EGFP, which drives the mammalian expression of YFV-17D NS1 as a transcriptional fusion to enhanced green fluorescent protein (EGFP) was generated by PCR cloning of YFV-17D nt 2381 to 3508 cDNA (including an E protein-derived N-terminal signal peptide) (63) flanked by a 5′-terminal Kozak sequence and 3′-terminal stop codon into the NheI and SalI sites of pIRES2-EGFP (Clontech catalog no. 6029-1).

An MHC class I-restricted peptide from YFV-17D nonstructural protein 3 (NS3) (sequence ATLTYRML) (64) was synthetized by Eurogentec (Seraing, Belgium). Total cellular antigen for YFV-17D and JEV SA14-14-2 was prepared first by infecting Vero E6 cells with YFV-17D and JEV SA14-14-2, respectively, at a multiplicity of infection (MOI) of 0.1. Noninfected Vero E6 cells were used as a control. Four days postinfection, cells were harvested either by trypsinization or by detaching through pipetting when CPE was visible. Following centrifugation, cell pellets were resuspended in phosphate-buffered saline (PBS) and cell lysates were prepared by four freeze-thaw cycles. Overnight UV irradiation was performed to inactivate the virus in the cell lysate preparations, and large debris was removed via filtering through 70-μm cell strainers (BD Biosciences).

### Animals, hyperimmune serum, infection, and T cell depletions.

Host IFN signaling is the major barrier to viscerotropism and for pathogenicity of neurotropic flaviviruses (65). In line, wild-type mice are poorly susceptible to infection with flaviviruses (28, 66–68), including YFV-17D infection and vaccination (34, 35, 65). Likewise, type I (IFN-α/β) and type II (IFN-γ) interferon signaling mutually controls YFV-17D infection. Unlike in humans (69, 70), type I IFN (IFN-α/β) can restrict YFV-Asibi as well as YFV-17D infection in mice (34, 35, 71, 72). Similarly, IFN-γ exerts restriction on YFV-17D replication, dissemination, and clearance in mice (35, 73). YFV-17D is neurotropic in wt mice when directly injected into the brain (52). In AG129 mice, IFN-α/β and -γ receptors are knocked out, which results in neurotropic infection following peripheral inoculation of YFV-17D. Therefore, to allow transient replication (and thus vaccination) of YFV-17D in wt mice, MAR1-5A3 antibodies were coadministered to block type I IFN signaling in C57BL/6 mice.

Immunodeficient IFN-α/β and -γ receptor knockout mice (AG129; B&K Universal, Marshall BioResources, UK) were bred in-house. AG129 mice have been shown to be highly susceptible to lethal YFV-17D infection, serving as a well-established surrogate rodent challenge model for wt YFV infection (34, 35, 74). Six- to 8-week-old male AG129 mice were used for all experiments after random assignment into different groups. Animals were kept in individually ventilated type-2 filter top cages on a 12-h/12-h day/night cycle with water and food *ad libitum*. Housing of animals and procedures involving animal experimentation were conducted in accordance with institutional guidelines approved by the Ethical Committee of the KU Leuven, Belgium (licenses P168/2012, P103/2015, P140/2016, and P005/2018). Throughout the study, animals were vaccinated intraperitoneally (i.p.) with either 10^4^ PFU of JE-CVax, 1/6th human dose of Ixiaro (25), or 2% assay medium. Animals vaccinated with Ixiaro were boosted on 14 dpv with 1/6th human dose of Ixiaro. All the vaccinated animals were challenged i.p. with 10^3^ PFU of either YFV-17D or JEV SA14-14-2 (both corresponding to 1,000 LD_50_) 28 dpv, if not stated otherwise. An additional 4 weeks after YFV-17D challenge, some animals were rechallenged i.p. with 10^4^ PFU of ZIKV-MR776 (60). Animals were observed for morbidity (weight loss) and humane endpoint once daily. The humane endpoint is defined as either paresis/difficulty in walking, paralysis (hind legs/soured eyes), moribundity/ataxia/tremors/difficulty in breathing, 20% weight loss, or quick weight loss (15% within 1 or 2 days); animals were immediately euthanized once a humane endpoint was reached. Throughout the study, bleedings were performed through submandibular puncture on day 0 (prevaccination), day 28 (postvaccination), and day 56 (postchallenge).

Hyperimmune serum was prepared by vaccinating AG129 mice with 10^4^ PFU of JE-CVax, followed by two boosts with 10^5^ PFU of JE-CVax at 14-day intervals. Another 14 days after the second booster, animals were bled twice per week for the following 4 weeks. All serum batches were then pooled and CPENTs for JE-CVax and YFV-17D were performed. We did not observe any YFV nAbs in the hyperimmune sera but did see an ∼3.6-fold (log_10_ CPENT_50_ titer, 3.03 ± 0.18) selective increase in neutralizing titers against JE-CVax compared to single vaccination (Fig. 3A). Normal mouse control serum was prepared by pooling serum from 18 nonvaccinated AG129 mice.

Immunocompetent wt C57BL/6JOIaHsd, i.e., C57BL/6, mice were purchased from ENVIGO Labs, the Netherlands, and were maintained and manipulated as described for AG129 mice, with some modifications (23, 28). Since flaviviruses do not readily replicate in immunocompetent wild-type mice (28), the mice were immunized with 10^4^ PFU of JE-CVax in the presence of 2.5 mg of an IFN-α/β receptor subunit 1 (IFNAR-1) binding monoclonal antibody, MAR1-5A3, administered i.p. 1 day prior to immunization. MAR1-5A3 antibody (0.5 mg) was also readministered i.p. on day 4 and day 7 postvaccination. Animals were bled 28 days postvaccination and challenged i.c. with 10^4^ PFU of YFV-17D in a volume of 30 μl. A full characterization of immunogenicity of YFV-17D in various mouse strains is available upon request (J. Ma, N. Mishra, R. Boudewijns, J. Neyts, and K. Dallmeier, unpublished data). For T cell depletion studies, C57BL/6 mice were either sham vaccinated or vaccinated i.p. with 1 × 10^4^ PFU of JE-CVax 35 days prior to i.c. challenge with 1 × 10^4^ PFU of YFV-17D. At day −2 and day 0 prior to YFV challenge, 0.5 mg of either anti-mouse CD4 (clone GK1.5; Leinco Technologies, USA) or anti-mouse CD8a (clone 53-6.7; Leinco Technologies) or a combination of both was administered i.p. (32, 75).

### IIFA.

To determine the seroconversion of animals, all JEV, YFV, and ZIKV IgG indirect immunofluorescence assays (IIFAs) were performed as per the manufacturer’s instructions (Euroimmun, Lübeck, Germany), except for the use of labeled secondary antibody and the mounting agent glycerine, which were replaced by Alexa Fluor 488 goat anti-mouse IgG (A-11029; Thermo Fisher Scientific) and 4′,6-diamidino-2-phenylindole (DAPI; ProLong antifade reagent with DAPI; Thermo Fisher Scientific), respectively. Serum from nonvaccinated animals served as a naive, negative control. Slides were visualized using a fluorescence microscope (FLoid cell imaging station; Thermo Fisher Scientific).

### Plaque assay and plaque reduction neutralization testing (PRNT).

Viral titers of YFV-17D or JE-CVax preparations were determined using plaque assays on BHK-21J cells. In brief, 10^6^ BHK-21J cells per well were plated in 6-well plates and cultured overnight in seeding medium. Cells were washed with PBS and inoculated with virus of different dilutions prepared in the assay medium for 1 h at room temperature (RT). Culture supernatants of uninfected cells were used as negative controls. Cells were thoroughly washed with the assay medium and overlaid with 2× minimal essential medium (MEM; Gibco, Belgium) supplemented with 4% FCS and 0.75% sodium bicarbonate containing 0.5% low-melting-point agarose (Invitrogen, USA). The overlay was allowed to solidify at RT; cells were then cultured for 7 days at 37°C, fixed with 8% formaldehyde, and stained with methylene blue. Plaques were manually counted and plaque titer was determined as PFU per milliliter.

Throughout the study, all the virus neutralization assays, i.e., PRNT and CPE-based virus neutralization testing (CPENT), were performed with YFV-17D and JE-CVax. JE-CVax has previously been established as a safe substitute for JEV, a BSL3 pathogen, when virus neutralization tests need to be performed at BSL2 (76). PRNT was performed similarly to the plaque assays for viral titration, with some modifications. Briefly, a step was added, in which different serum dilutions made in the assay medium were first inoculated with YFV-17D (20 to 50 PFU) or JE-CVax (50 to 100 PFU) virus for 1 h at 37°C and then added to the cells. All sera were assayed in triplicate in serial dilutions 1:20, 1:66, 1:200, 1:660, 1:2,000, and 1:6,600. Plaques were manually counted and PRNT_50_ were calculated using the Reed and Muench method (77). Culture-derived YFV-17D and JE-CVax were used as positive virus controls, while culture supernatants of uninfected cells were used as a negative cell control. PRNT_50_ values for each sample represent geometric means of three independent repeats, and data are presented as log_10_ PRNT_50_ (mean ± standard deviation [SD]).

### CPE assay and CPENT.

Viral titers for culture-derived YFV-17D or JE-CVax (TCID_50_) and 50% neutralizing antibody titers (log_10_ CPENT_50_) were determined using CPE-based cell assays and CPE-based virus neutralization tests, respectively, on BHK-21J cells as described previously (78), with some modifications. In brief, 2 × 10^4^ BHK-21J cells/well were plated in 96-well plates overnight in seeding medium. The medium was then replaced with assay media containing different virus dilutions and cultured for 5 days at 37°C. Later, assays were first visually scored for CPE and then stained with MTS/phenazine methosulphate (PMS; Sigma-Aldrich) solution for 1.5 h at 37°C in the dark. Post-MTS/PMS staining, absorbance was measured at 498 nm for each well. All assays were performed in six replicates, and TCID_50_ per milliliter was determined using the Reed and Muench method (77).

CPENTs were performed similarly to the CPE assays for viral titration, with some modifications. Briefly, a step was added, in which different serum dilutions made in the assay medium were first inoculated with 100 TCID_50_ of YFV-17D or JE-CVax virus for 1 h at 37°C and then added to the cells. All sera were assayed in triplicate in serial dilutions 1:20, 1:66, 1:200, 1:660, 1:2,000, and 1:6,600. CPE neutralization was calculated with the following formula: percent neutralization activity = percent CPE reduction = (OD_virus+serum_ – OD_VC_) × 100/(OD_CC_ – OD_VC_), where OD is optical density; 50% neutralization titers (CPENT_50_) were calculated using the Reed and Muench method (77). Culture-derived YFV-17D and JE-CVax were used as positive virus controls (VC), while culture supernatants of uninfected cells were used as a negative cell control (CC). CPENT_50_ values for each sample represent geometric means of three independent repeats, and data are presented as log_10_ CPENT_50_ (mean ± SD). CPENT for detection of nAbs was validated against a standard PRNT, yielding a strong correlation (*R*^2^ = 0.71; *P* = 0.018) between PRNT_50_ and CPENT_50_ (Fig. S8A) and similar anti-JEV nAb titers in postvaccination and postchallenge serum samples (Fig. S8B and C).

10.1128/mBio.02494-19.8 FIG S8 Correlation of nAb titers determined as log_10_ PRNT_50_ and log_10_ CPENT_50_ in matched serum samples of JE-CVax-vaccinated and/or YFV-17D-challenged mice. AG129 mice (*n* = 7) were vaccinated with 10^4^ PFU of JE-CVax and at 28 dpv challenged with 10^3^ PFU YFV-17D. Serum was harvested and neutralization assays, i.e., CPENT and PRNT, were performed as described in Materials and Methods. Data show a good correlation (Pearson correlation [*R*^2^] = 0.071; *P* = 0.02) between log_10_ YFV PRNT_50_ and log_10_ YFV CPENT_50_ of matched samples (A). There was no marked increase in the log_10_ JE-CVax PRNT_50_ and log_10_ JE-CVax CPENT_50_ (*P* values, 0.156 and 0.062, respectively) found when comparing matched serum samples from before and after challenge with YFV-17D using Wilcoxon matched-pairs signed rank test (B and C). The limit of detection for each assay was log_10_20, i.e., 1.3. Download FIG S8, JPG file, 0.2 MB. Copyright © 2020 Mishra et al. 2020 Mishra et al. This content is distributed under the terms of the Creative Commons Attribution 4.0 International license.

### ELISA.

Serum antibodies recognizing YFV NS1 were detected by indirect enzyme-linked immunosorbent assay (ELISA), in essence as previously described (75, 76, 80). In brief, ELISA plates (Nunc MaxiSorp; Thermo Fisher Scientific) were coated with 1 μg/ml of recombinant YFV NS1 (Bio-Rad; catalog no. PIP052A) in 50 mM carbonate buffer (pH 9.6) overnight at 4°C. After three washes with PBS-T (PBS with 0.05% Tween 80), plates were blocked with 2% bovine serum albumin (BSA) in PBS-T for 1 h at 37°C or overnight at 4°C. After three washes with PBS-T, wells were treated with serial dilutions of test sera (2-fold serial dilution in PBS-T) for 2 h at room temperature. Serial dilutions of the YFV NS1-specific mouse IgG2a monoclonal antibody (clone 1A5, kindly provided by J. J. Schlesinger) (27) starting at 10 μg/ml served as a standard. After four washes with PBS, plates were incubated with horseradish peroxidase-labeled goat anti-mouse IgG antibody (Sigma-Aldrich; catalog no. AP124P; diluted 1:3,000 in PBS-T) for 1 h. After another four washes with PBS, bound antibodies were detected via conversion of added tetramethylbenzidine (TMB; SureBlue TMB microwell peroxidase; KPL). The reaction was stopped after 10 min by adding equal quantities of 1 M HCl solution, and absorbance was measured at 450 nm. After background subtraction, relative anti-YFV NS1 titers were determined by comparison to the standard curve generated for MAb 1A5 included in each assay plate. To that end, the dilution at which each individual test serum yielded an OD at 450 nm (OD_450_) of 1 was used to calculate an absolute anti-NS1 antibody concentration (equivalent concentration), assuming a binding similar to that of YFV-17D NS1 by MAb 1A5. Only values that exceeded three times the background signal were considered positive.

### ADCC bioassay.

To assess the possible role of nonneutralizing YFV antibody-mediated protection against YFV after JE-CVax vaccination, antibody-dependent cell-mediated cytotoxicity (ADCC) bioassays (29) were performed as prescribed by the manufacturer (ADCC reporter bioassay complete kit; Promega; catalog no. G7010). In brief, target cells were prepared by infecting HEK 293T cells with YFV-17D-mCherry in assay medium. Cells were incubated at 37°C postinfection and later, upon onset of CPE, harvested by trypsinization. Cells were again plated in white, flat-bottom 96-well assay plates (Viewplate-96; PerkinElmer; catalog no. 6005181) at densities of 7,500 and 25,000 cells per well for 8 h at 37°C in assay medium. Later, in a separate 96-well plate, JE-CVax hyperimmune and nonimmune heat-inactivated mouse serum samples (starting dilution of 1/9) were serially diluted 3-fold in RPMI 1640 medium (Gibco, Thermo Fisher Scientific) supplemented with 4% low-IgG serum. ADCC bioassay effector cells (Jurkat V variant cells) were diluted to 3 × 10^6^ cells/ml in RPMI 1640 medium. The supernatant from the infected cell plate was replaced with fresh RPMI medium (25 μl/well), and diluted serum samples (25 μl/well) and E cells (25 μl/well) were added to the infection plate. After incubation at 37°C for 24 h, Bio-Glo luciferase assay reagent (75 μl/well) was added, and luminescence was measured using a Spark multimode microplate reader (Tecan). The average background plus three SDs was calculated and used as background.

### Intracellular staining of NS1 protein in HEK 293 cells.

HEK 293 cells transfected with pCMV-YFV-17D NS1-IRES-EGFP or infected with YFV-17D-mCherry were detached with trypsin-EDTA (0,05%), centrifuged (at 2,500 rpm and 4°C for 5 min), and suspended in FACS-B (Dulbecco's phosphate-buffered saline [DPBS], no Ca^2+^/Mg^2+^, 2% fetal bovine serum [FBS], 2 mM EDTA). Not more than 5 × 10^6^ cells per well were seeded into round-bottom 96-well plates (Costar, Corning Inc.) and spun down, and the supernatant was removed. Dead cells were stained *in vitro* with ZombieAqua (Biolegend; 1:500 diluted in DPBS) to exclude from further analysis. After washing and fixation with 2% paraformaldehyde (in FACS-B), cells were permeabilized by 0.1% saponin (in FACS-B with 1% normal mouse serum) with streptavidin added (streptavidin/biotin blocking kit; Vector Laboratories) to block endogenous biotin (permeabilizing and blocking solution). The cells were then stained with anti-NS1 primary antibody solution (clone 1A5; 5 μg/ml), or JE-CVax-vaccinated mouse serum (1:10) in permeabilizing and blocking solution. Anti-NS1 antibody binding was detected by a biotinylated goat anti-mouse IgG secondary antibody solution (Thermo Fisher Scientific; catalog no. A16076; 1:200 dilution). The biotinylated secondary antibody was stained subsequently with streptavidin-phycoerythrin (PE)-Cy7 (Biolegend; 1:200 dilution). After the cells were washed, they were resuspended in FACS-B and filtered through 100-μm nylon meshes (Sefar; ELKO Filtering; 03-100/44) prior to analysis on a flow cytometer (LSR Fortessa X-20; Becton, Dickinson). The data were analyzed using FlowJo 10 software (TreeStar). The gating strategy for the analysis is depicted in Fig. S9A.

10.1128/mBio.02494-19.9 FIG S9 Gating strategy for flow cytometry analysis. (A and B) Exclusion of debris was achieved by gating out the FSC-low population in a plot of FSC-A versus SSC-A. Then, only single cells were retained by elimination of the high-SSC-W population in a plot of SSC-W versus SSC-H. In a subsequent step, live cells were selected by gating out the Zombie Aqua-positive population as shown in a plot of NS1-eGFP/FSC-A versus ZA. Finally, for the detection of anti-NS1 antibodies (A), cells were gated based on positivity for NS1-eGFP and positivity/negativity of anti-NS1 Ab PE-Cy7. (B) For intracellular cytokine staining, based on positivity for CD3e (eFluor450 conjugated) and negativity (CD4) or positivity for CD8a (APC/Cy7-conjugated), CD4^+^ and CD8^+^ T cell populations were defined as CD3^+^ CD8^−^ and CD3^+^ CD8^+^ populations, respectively. Finally, cells were gated based on positivity/negativity for IFN-γ and positivity/negativity of TNF-α. Samples from nonvaccinated mice were used to set the boundaries that define cells positive and negative for intracellular markers. Download FIG S9, JPG file, 0.5 MB. Copyright © 2020 Mishra et al. 2020 Mishra et al. This content is distributed under the terms of the Creative Commons Attribution 4.0 International license.

10.1128/mBio.02494-19.10 FIG S10 Binding predictions for peptides (8- to 14-mers) comprised in the YF-17D nonstructural proteins for mouse, human, and rhesus macaque MHC classI. For the 20 strongest binding peptides (lowest dissociation constant [*K_d_*]) for each haplotype, the position of the first amino acid in the NS proteins is given, as well as the affinity of the peptide for the MHCI. HLA-binding peptides indicated in colors are (or at least contain) YFV-17D-derived peptides that have been experimentally confirmed by Lund (O. Lund, PloS One 6:e26494, 2011, https://doi.org/10.1371/journal.pone.0026494) (blue), de Melo et al. 2013 (A. B. de Melo, E. J. M. Nascimento, U. Braga-Neto, R. Dhalia, A. M. Silva, M. Oelke, J. P. Schneck, J. Sidney, A. Sette, S. M. L. Montenegro, and E. T. A. Marques, PLoS Negl Trop Dis 7:e1938, 2013, https://doi.org/10.1371/journal.pntd.0001938) (yellow), Blom et al. (K. Blom, M. Braun, M. A. Ivarsson, V. D. Gonzalez, K. Falconer, M. Moll, H.-G. Ljunggren, J. Michaëlsson, and J. K. Sandberg, J Immunol 190:2150–2158, 2013, https://doi.org/10.4049/jimmunol.1202234) (red), and Kongsgaard et al. (M. Kongsgaard, M. R. Bassi, M. Rasmussen, K. Skjødt, S. Thybo, M. Gabriel, M. B. Hansen, J. P. Christensen, A. R. Thomsen, S. Buus, and A. Stryhn, Sci Rep 7:1–14, 2017, https://doi.org/10.1038/s41598-017-00798-1) (green). Download FIG S10, JPG file, 0.2 MB. Copyright © 2020 Mishra et al. 2020 Mishra et al. This content is distributed under the terms of the Creative Commons Attribution 4.0 International license.

### Processing of mouse spleens for the preparation of single-cell suspensions.

Six- to 8-week-old C57BL/6 or AG129 mice were vaccinated with 10^4^ PFU of JE-CVax and 4 and 18 weeks later, the animals were euthanized for analysis. Spleens were harvested and processed for ELISpot and flow cytometric analyses. To generate single-cell suspensions, spleens were pushed through 70-μm cell strainers (BD Biosciences) with syringe plungers, digested in 1.0 mg/ml of type 1 collagenase and 10 U/ml DNase for 30 min, vigorously pipetted, and filtered through 100-μm nylon meshes. Spleen samples were then incubated with red blood cell lysis buffer (eBioscience) for 8 min at room temperature and washed twice with FACS-B.

### ELISpot assay.

Mouse TNF-α and IFN-γ enzyme-linked immunospot (ELISpot) assays were performed with a mouse TNF-α ELISpot kit (ImmunoSpot MTNFA-1M/5; CTL Europe GmbH) or a mouse IFN-γ ELISpot kit (ImmunoSpot MIFNG-1M/5; CTL Europe GmbH) according to the manufacturer’s instructions. Assay plates (96-well polyvinylidene difluoride [PVDF] membrane), antibodies, enzymes, substrate, and diluent were included in the kits. Briefly, 4 × 10^5^ mouse splenocytes/well were plated with either with 5 μg/ml of YFV-17D NS3 ATLTYRML peptide antigen (60) or with 50 μg/ml of total Vero E6 cellular antigen in RPMI 1640 medium (Gibco, Belgium) supplemented with 10% fetal bovine serum, 2 mM l-glutamine, and 0.75% sodium bicarbonate. After 24 h of incubation at 37°C, spots of mouse TNF-α or IFN-γ were visualized by subsequent addition of detection antibody, enzyme, and substrate. All plates were scanned using an ImmunoSpot S6 universal reader (CTL Europe GmbH). Spot counts were normalized by subtracting the number of spots from corresponding samples stimulated with noninfected Vero E6.

### Intracellular cytokine staining for memory T cells and flow cytometry.

To restimulate memory T cells, freshly isolated single cell suspensions of splenocytes were seeded at 3 × 10^6^ per well in a round-bottom 96-well plate and incubated with either 5 μg/ml of the MHC class I- restricted peptide from YFV-17D NS3 (ATLTYRML) (60) or 50 μg/ml of total cellular antigen (infected or noninfected Vero E6 cell lysate). Following overnight incubation, the splenocytes were incubated for 2 h with 5 μg/ml of brefeldin A (Biolegend) for intracellular trapping of cytokines and then stained with Zombie Aqua (1:200) in PBS for 15 min. Splenocytes were then stained for CD3 (4 μg/ml eFluor 450 α-mouse CD3 antibody; Thermo Fisher Scientific) and CD8 (2 μg/ml of allophycocyanin [APC]/Cy7 α-mouse CD8a antibody; Biolegend) in PBS for 20 min before fixation in 2% paraformaldehyde (Sigma-Aldrich) and permeabilization and blocking in a mixture of 0.1% saponin and 1% normal mouse serum. Finally, splenocytes were stained intracellularly for TNF-α (6.5 μg/ml PE anti-mouse TNF-α; Biolegend) and IFN-γ (2 μg/ml of APC anti-mouse IFN-γ; Biolegend) prior to analysis on a flow cytometer (LSR Fortessa X-20; Becton, Dickinson). Gating on forward scatter A (FSC-A)/side scatter A (SSC-A) excluded debris; SSC-H/SSC-W and FSC-H/FSC-W excluded doublet cells. The data were analyzed using FlowJo 10 software (TreeStar). To determine the percentage of responding CD4^+^ or CD8^+^ T lymphocytes, the percentages of responders from samples stimulated with noninfected Vero E6 cell lysates were subtracted from the corresponding responses. The gating strategy for the analysis is depicted in Fig. S9B.

### Binding prediction for cytotoxic T lymphocyte (CTL) epitopes within the YFV-17D nonstructural proteins.

Binding to the MHC class I molecules of peptides comprised in the NS proteins of YFV-17D was predicted by NetMHCPan4.0 (http://www.cbs.dtu.dk/services/NetMHCpan/). The full amino acid sequence of YFV-17D NS proteins was entered into the server to predict the 20 peptides (8- to 14-mers) with the highest affinity to their respective MHC/HLA class I molecules, such as mouse H2-Kb (C57BL/6 and S129 background), rhesus macaque Mamu-A*01, and some commonly found HLA types (HLA-A*02, HLA-A*24, and HLA-B*15) in men (79).

### Statistical analysis.

GraphPad Prism 7 (GraphPad Software, Inc.) was used for all statistical evaluations. Quantitative data were represented as means ± standard deviations and obtained from at least three independent experiments. For ADCC assays, flow cytometry analysis and ELISpot assay data were represented as means ± standard errors of means (SEMs). Statistical significance was determined using survival analysis with log rank (Mantel-Cox) test, one-way analysis of variance (ANOVA) (neutralization titers and ELISA), two-way ANOVA (ADCC), paired *t* test (flow cytometry), and Wilcoxon matched-pairs signed rank test (comparison of paired postvaccinated and postchallenge samples). Correlation studies were performed using linear regression analysis with Pearson’s correlation coefficient. Values were considered statistically significantly different at *P* values of ≤0.05.
