# Supplementary figures and images for: A Chimeric Japanese Encephalitis Vaccine Protects against Lethal Yellow Fever Virus Infection without Inducing Neutralizing Antibodies
Source: mBio. 2020 Apr 7;11(2):e02494-19. doi: 10.1128/mBio.02494-19 (PMC7157777; doi:10.1128/mBio.02494-19)

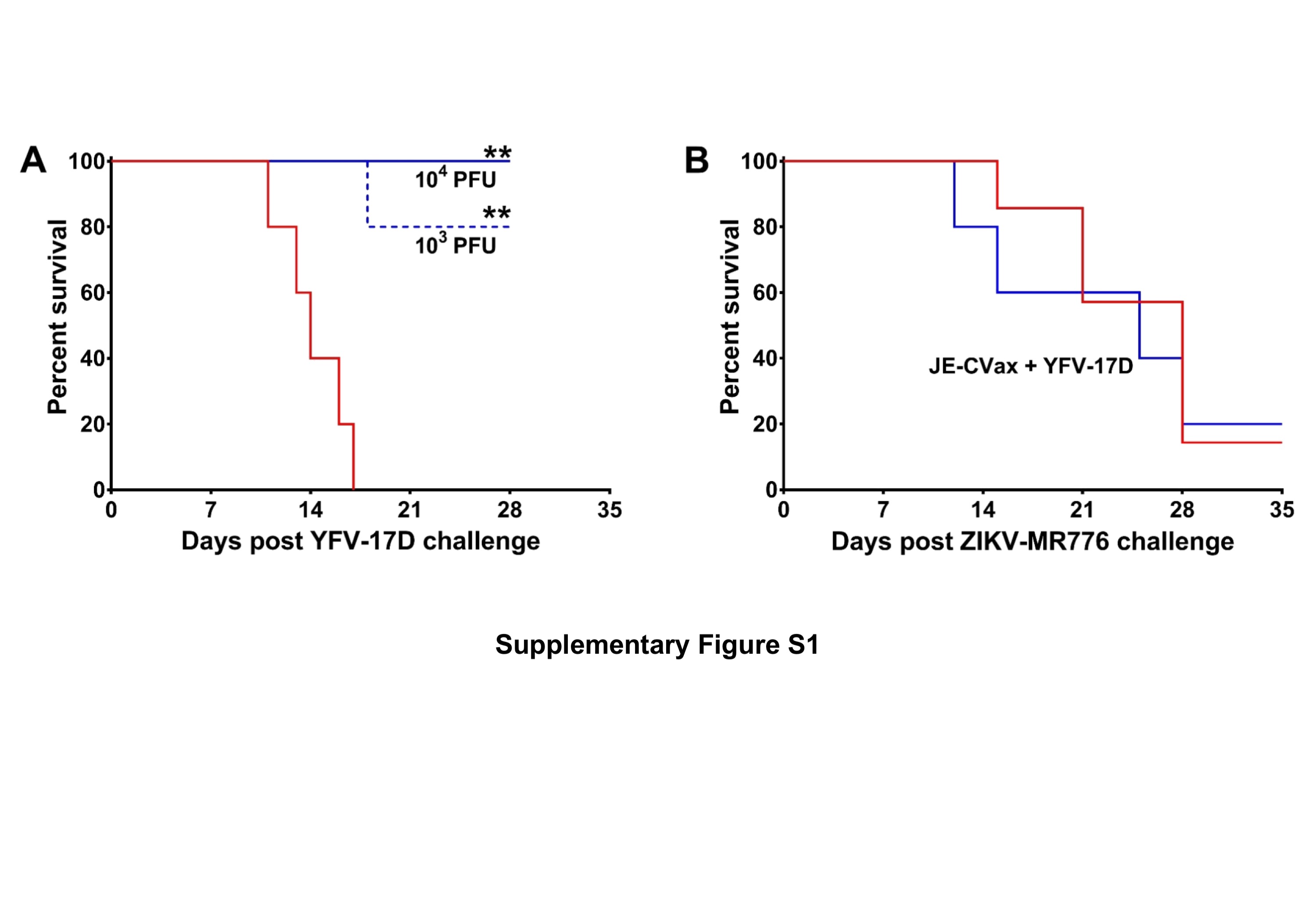

Supplement: FIG S1 [file mBio.02494-19-sf001.jpg]

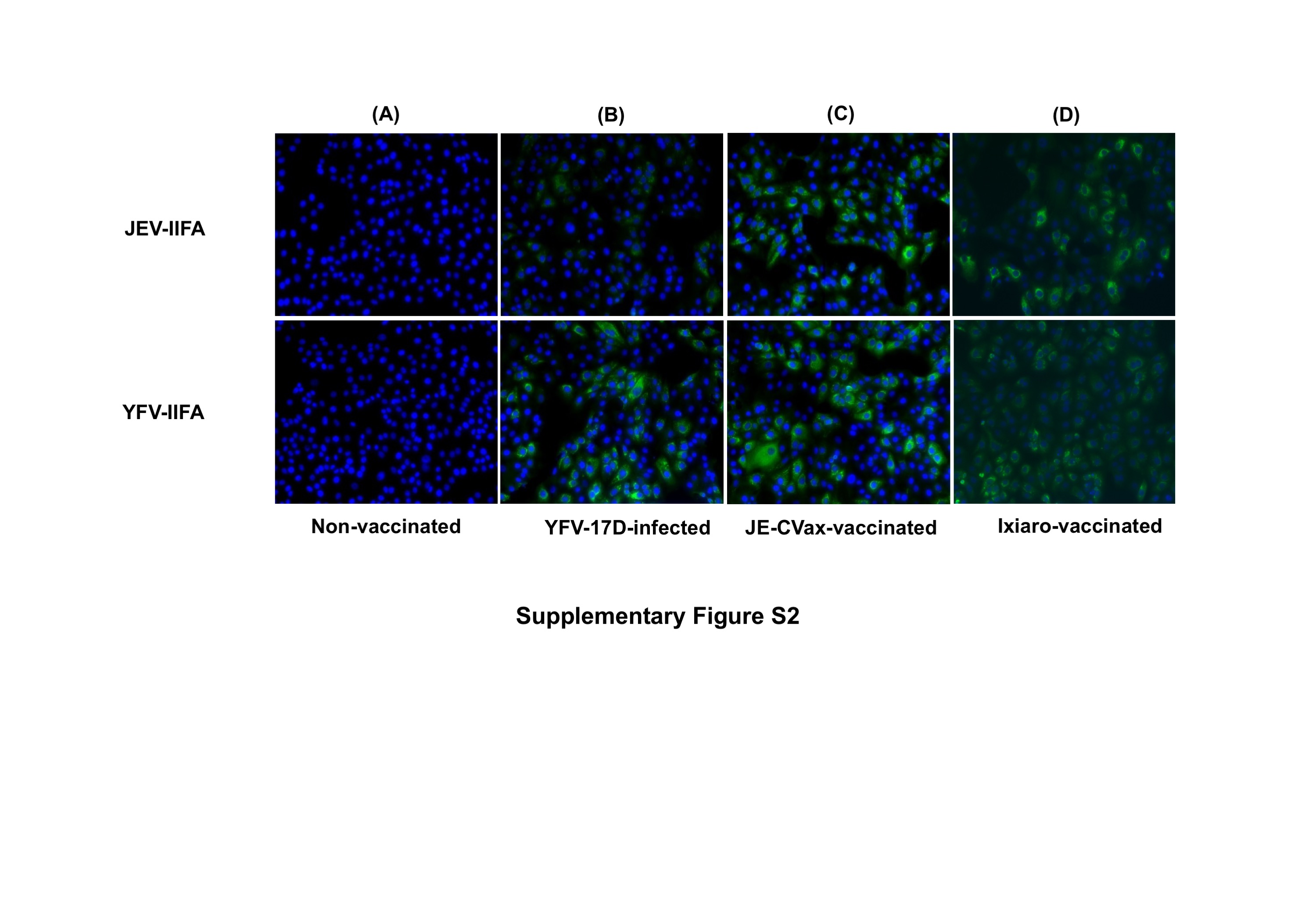

Supplement: FIG S2 [file mBio.02494-19-sf002.jpg]

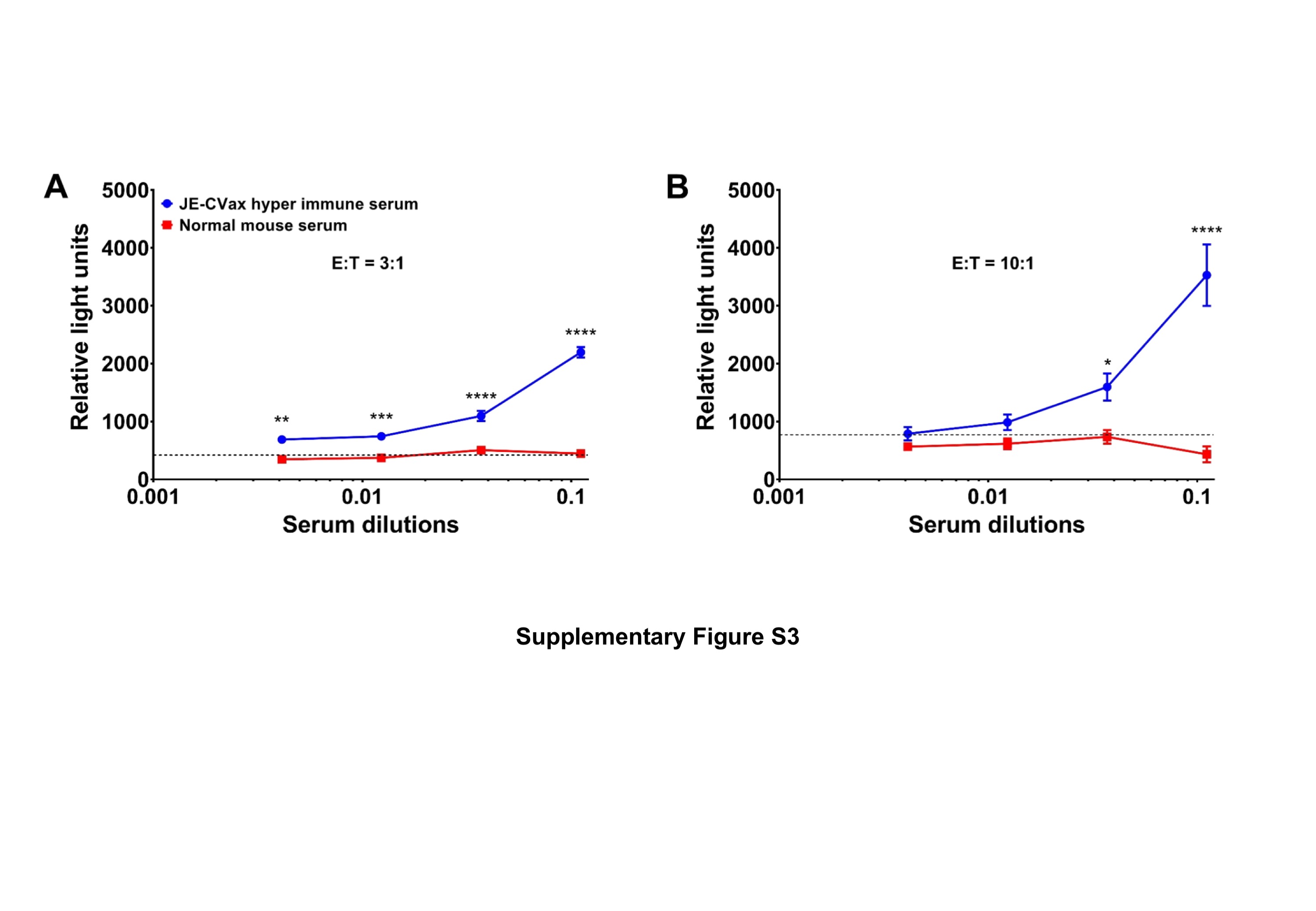

Supplement: FIG S3 [file mBio.02494-19-sf003.jpg]

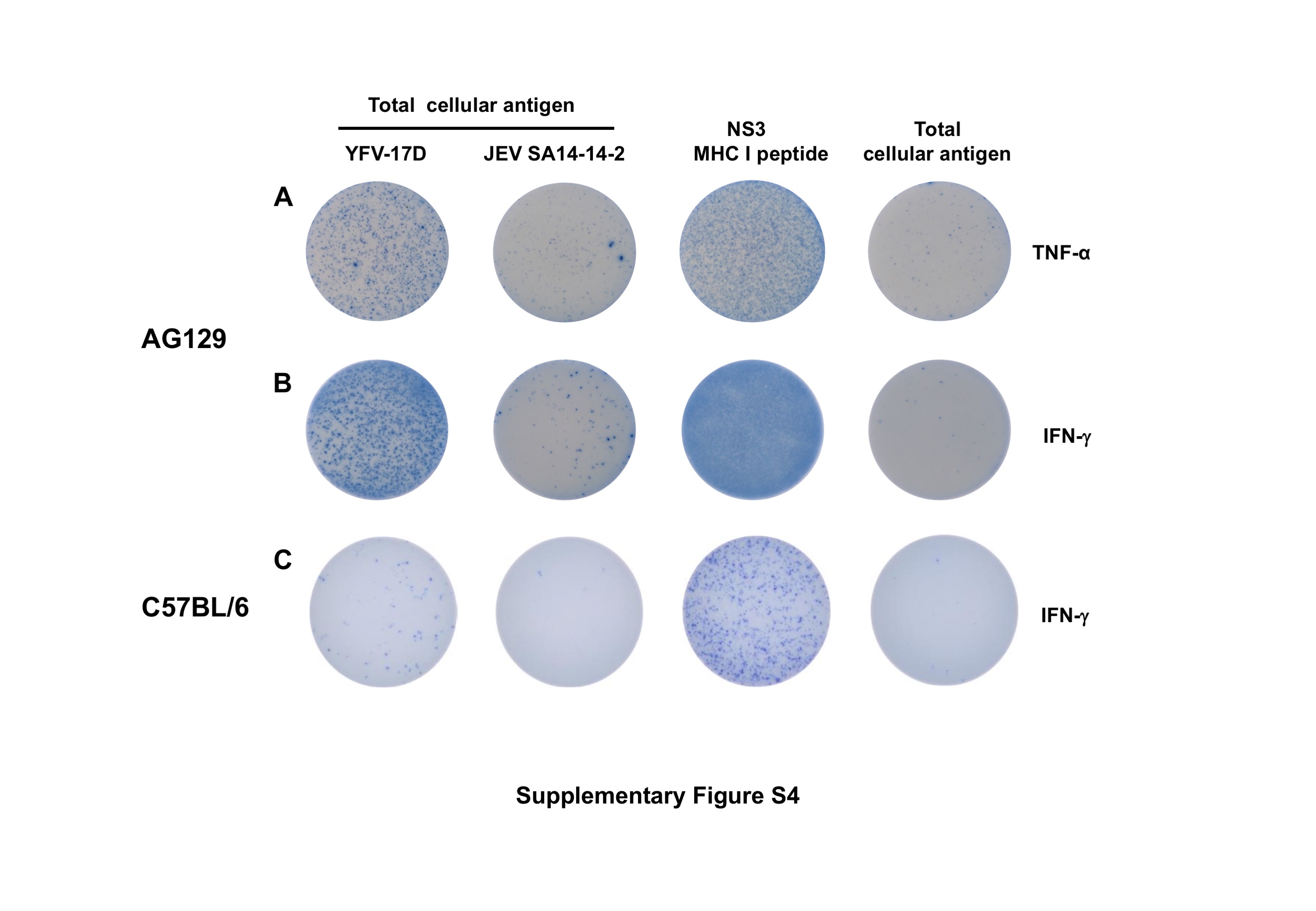

Supplement: FIG S4 [file mBio.02494-19-sf004.jpg]

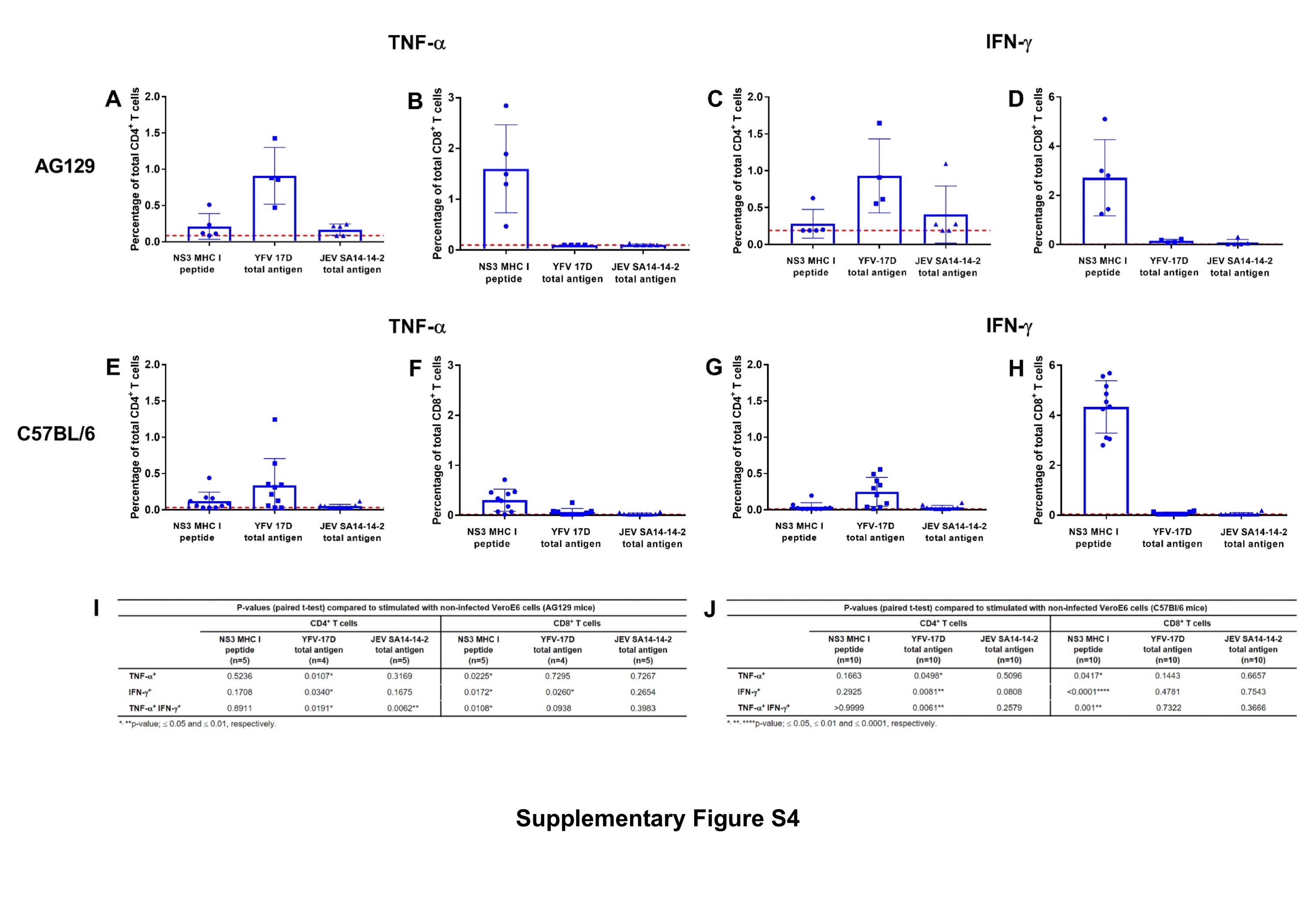

Supplement: FIG S5 [file mBio.02494-19-sf005.jpg]

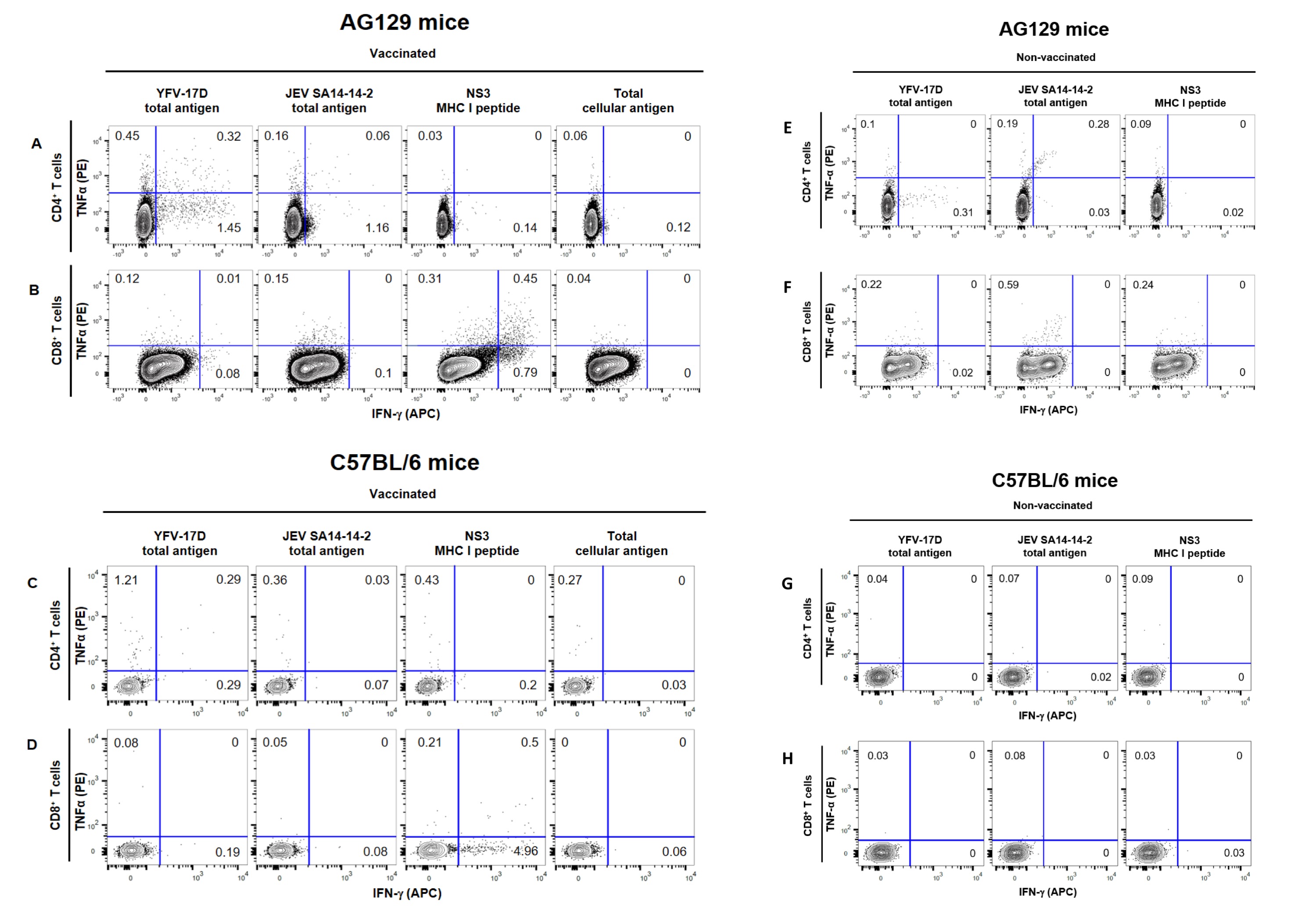

Supplement: FIG S6 [file mBio.02494-19-sf006.jpg]

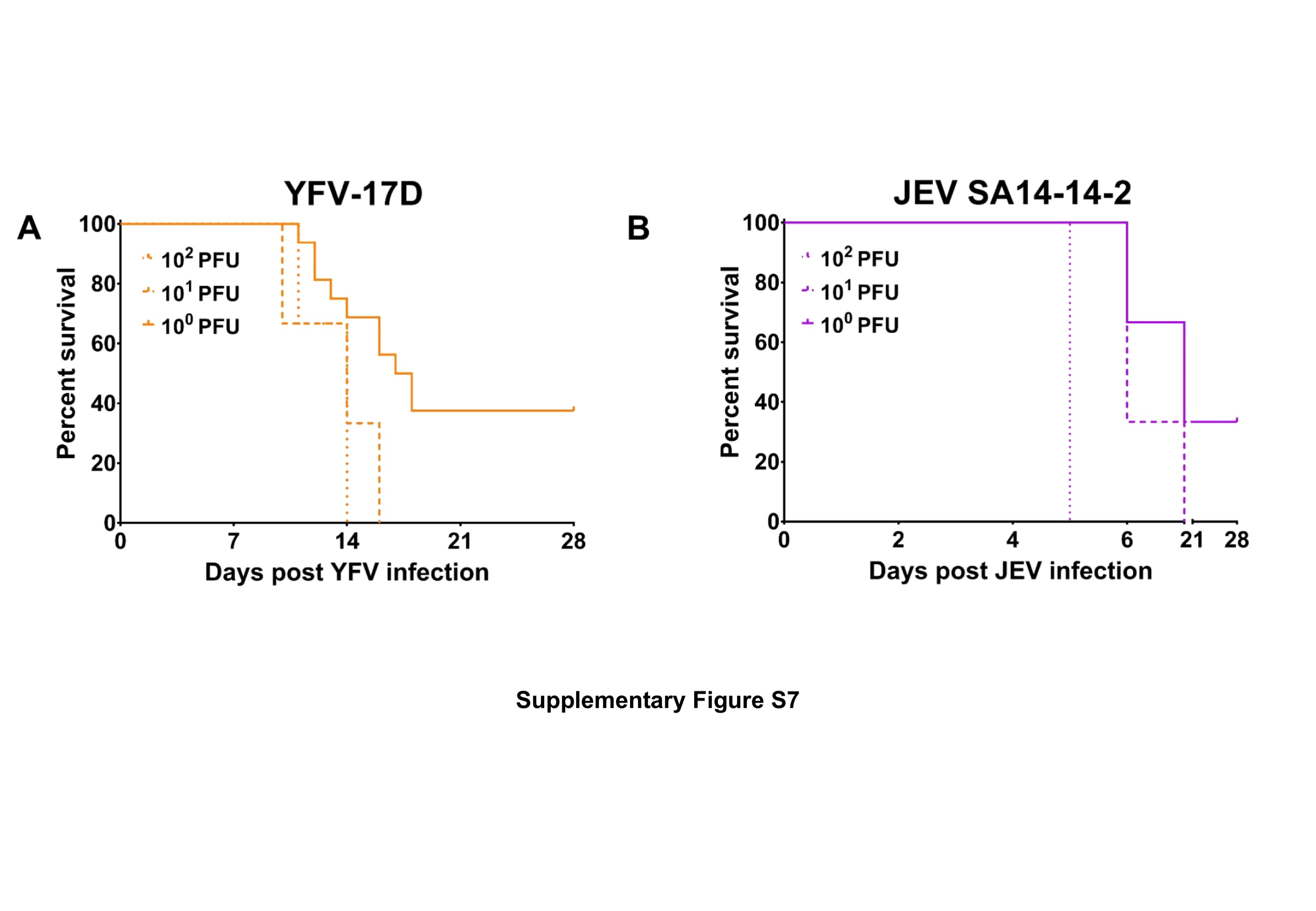

Supplement: FIG S7 [file mBio.02494-19-sf007.jpg]

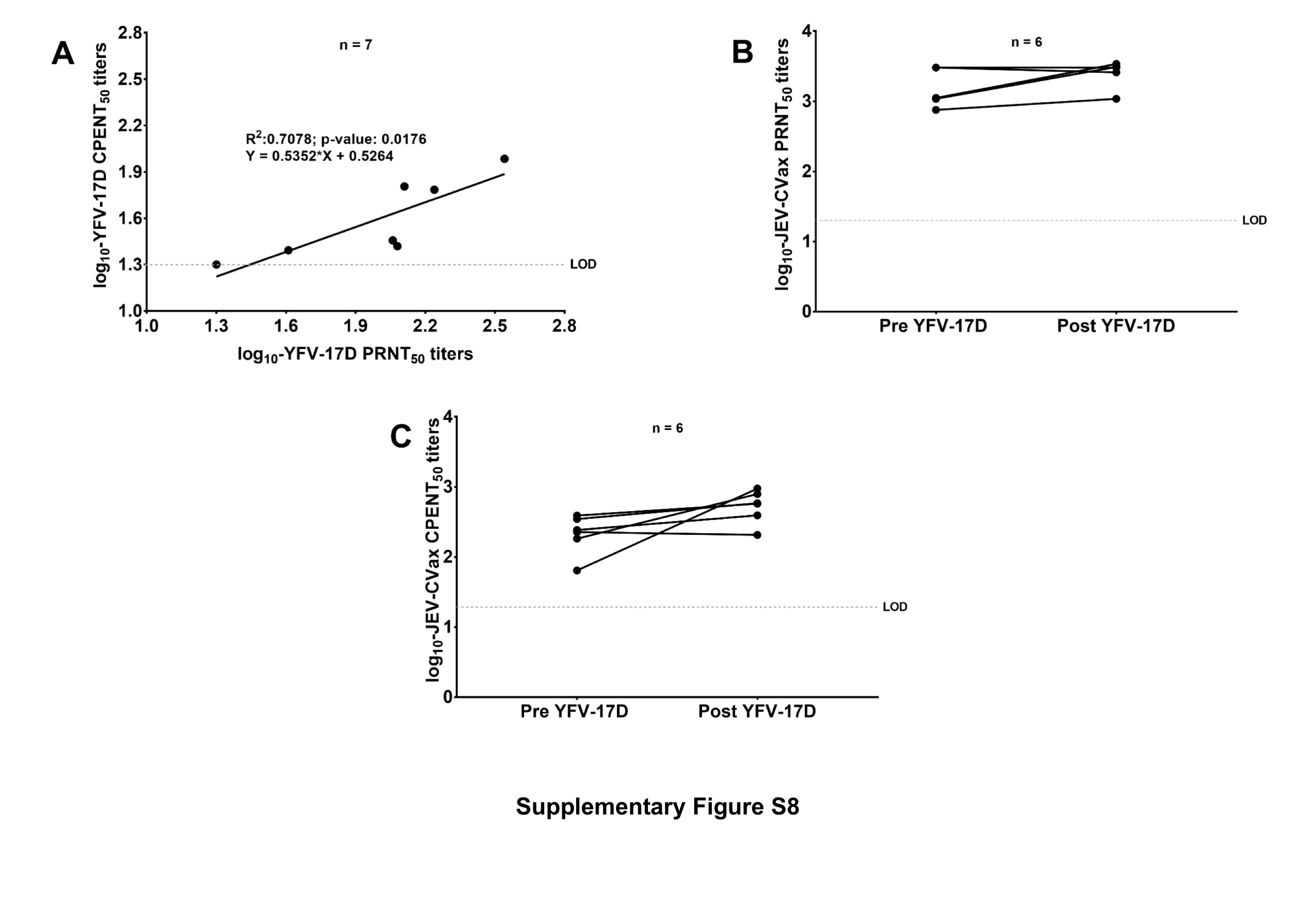

Supplement: FIG S8 [file mBio.02494-19-sf008.jpg]

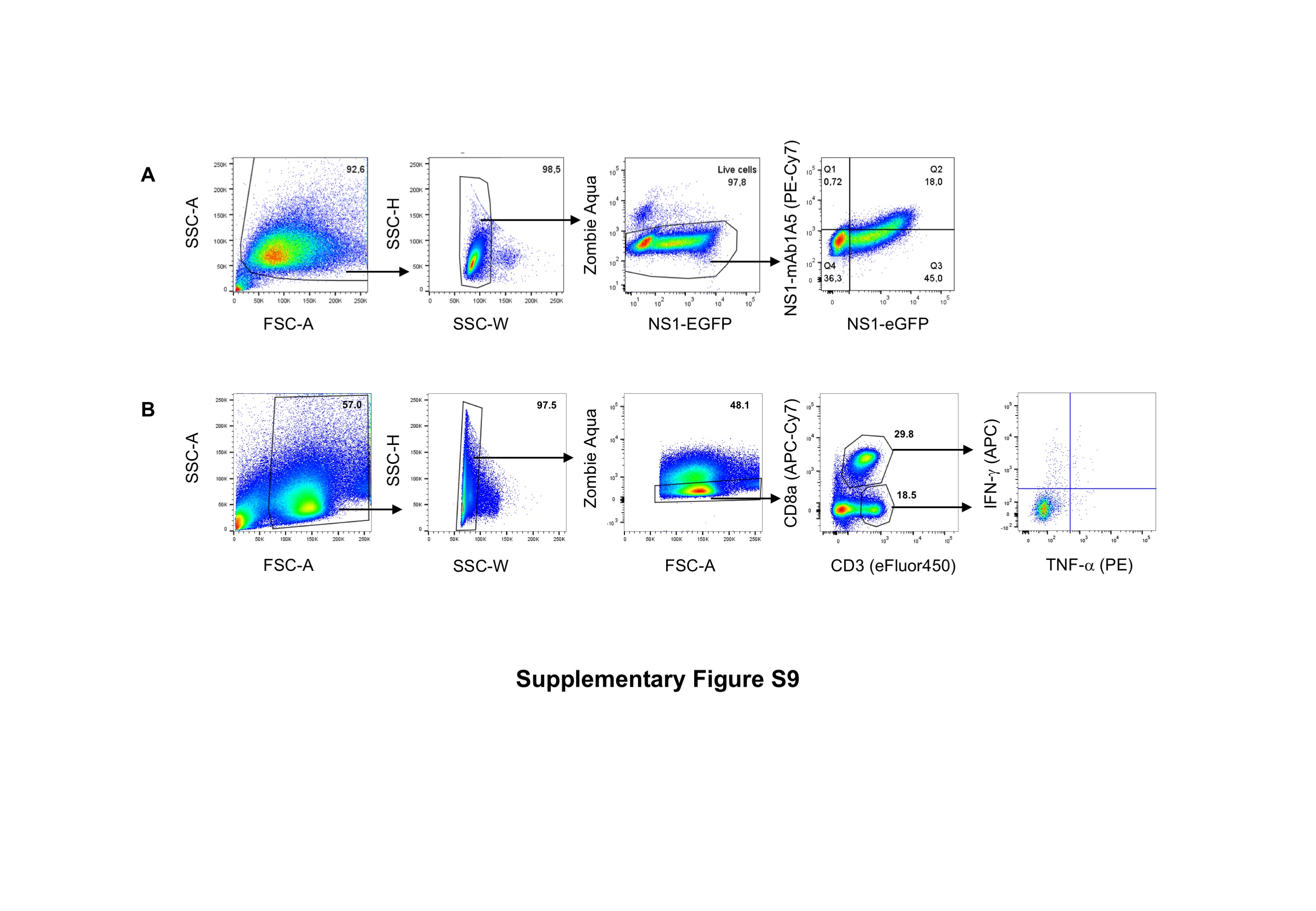

Supplement: FIG S9 [file mBio.02494-19-sf009.jpg]

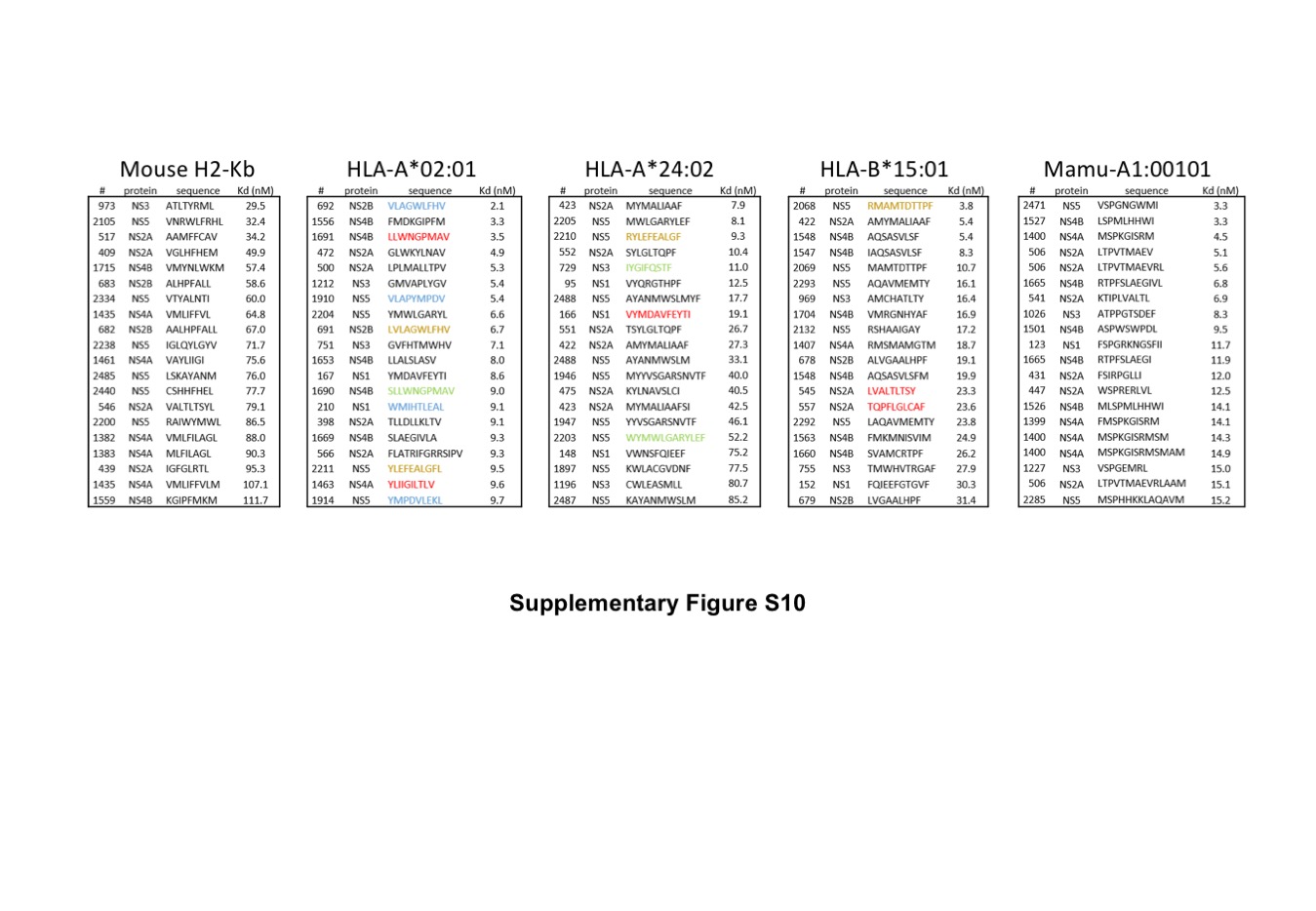

Supplement: FIG S10 [file mBio.02494-19-sf010.jpg]
